# Transcription–replication collisions trigger high-fidelity replication reset

**DOI:** 10.1093/nar/gkaf1227

**Published:** 2025-11-26

**Authors:** Matthew B Cooke, Kobie T Welch, Laura Deus Ramirez, Katelin M Hagstrom, Alice X Wen, Jennifer A Halliday, Susan M Rosenberg, Christophe Herman

**Affiliations:** Department of Molecular and Human Genetics, Baylor College of Medicine, Houston, TX 77030, United States; Department of Molecular and Human Genetics, Baylor College of Medicine, Houston, TX 77030, United States; Department of Molecular and Human Genetics, Baylor College of Medicine, Houston, TX 77030, United States; Department of Molecular and Human Genetics, Baylor College of Medicine, Houston, TX 77030, United States; Department of Molecular and Human Genetics, Baylor College of Medicine, Houston, TX 77030, United States; Department of Molecular and Human Genetics, Baylor College of Medicine, Houston, TX 77030, United States; Department of Molecular and Human Genetics, Baylor College of Medicine, Houston, TX 77030, United States; Dan L. Duncan Cancer Center, Baylor College of Medicine; Houston, TX 77030, United States; Department of Molecular Virology and Microbiology, Baylor College of Medicine, Houston, TX 77030, United States; Department of Molecular and Human Genetics, Baylor College of Medicine, Houston, TX 77030, United States; Dan L. Duncan Cancer Center, Baylor College of Medicine; Houston, TX 77030, United States; Department of Molecular Virology and Microbiology, Baylor College of Medicine, Houston, TX 77030, United States

## Abstract

Double-stranded DNA ends arise from external agents or cellular processes like transcription–replication collisions (TRCs), threatening genome stability. Here, we performed genomic CRISPRi screens to uncover DNA end formation factors in *Escherichia coli*. We discovered that translation–transcription decoupling causes DNA end formation through a TRC-dependent pathway, which is lethal when DNA end processing by RecBCD is disrupted, but not when recombination is disrupted. We find that TRCs cause replisome stalling followed by “rear-ending” from trailing replisomes which generates free DNA ends, rather than strand breaks. Surprisingly, these DNA ends are resolved through a process we call “replication reset”, where the stalled replicore is degraded, without triggering recombination, the DNA damage response, or mutagenesis. This hidden replicore-degradation resets the replication cycle without consequence for the genome. This discovery reveals a novel DNA safeguard mechanism for preserving genome stability when replication is disturbed and challenges the notion that TRCs necessarily cause genome instability in bacteria.

## Graphical Abstract

**Graphical Abstract:**
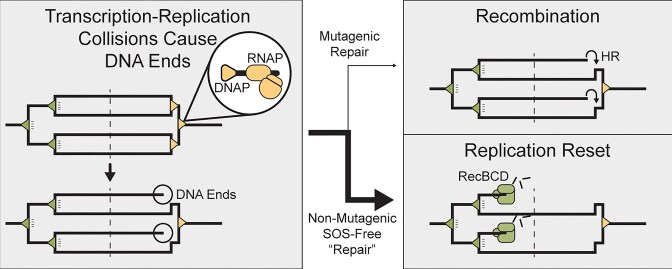

## Introduction

Spontaneous DNA double strand ends come from multiple endogenous cellular sources, threatening genomic integrity [1, 2]. These DNA ends are formed by internal processes such as replication stress [3]. Replication stress, particularly stress that causes stalled or collapsed replication forks, is a potent DNA end source [4]. Factors that may contribute to this replication stress include reactive oxygen species, topoisomerase malfunction, the formation of RNA–DNA hybrids, and transcription–replication collisions (TRCs) [5]. Recent genomic studies, using ChIP-seq and END-seq analysis, have provided insights into the replication-dependent nature of these breaks, particularly at specific genomic sites such as replication termini and sites of decatenation [5–7], but the contribution of TRCs to replication-dependent DNA ends is unclear.

Of the factors that contribute to replication stress and spontaneous DNA ends, TRCs are suggested to play a causal role [8–10]. TRCs are a common phenomenon, as the replication fork must pass through many active transcription units during each replication cycle. In most cases, the replisome clears these barriers with the assistance of the replisome-associate helicase, Rep [11]. However, in a subset of cases, these TRCs appear to result in toxic outcomes. A particularly toxic form of TRCs are thought to be driven by transcriptional disruptions that induce RNA polymerase pausing or backtracking, thereby increasing the likelihood of collisions between the transcription and replication machineries [12–14]. These states mediate toxic interactions with the DNA replication fork [9]. In *Escherichia coli*, these disruptions are mitigated by transcription-translation coupling [15], where the ribosome helps RNA polymerase avoid these stalled states [16, 17]. When coupling is lost, RNA polymerase becomes prone to forming stable, backtracked complexes, creating obstacles for the DNA replisome, leading to genotoxic TRCs. These toxic TRCs generate DNA ends which are thought to be repaired through homologous recombination by RecBCD [18], a repair pathway that can induce mutagenesis and genome rearrangements. Although TRC-induced DNA end formation is believed to accelerate genome evolution [19], the origin of these DNA ends remains elusive and their cellular consequences have largely been inferred.

Here, we use a DNA end binding protein, Gam from phage Mu, and a recombination null mutant, *ΔrecA*, both of which inhibit DNA end repair [2], to sensitize cells to DNA end formation in whole genome CRISPRi screens. Discrepancy between hits from these genome-wide screens identified a subset of gene knockdowns that promote DNA end formation without requiring *recA* for their repair. Most gene knockdowns in this *recA*-independent subset are involved in translation processing and transcription elongation. Our data reveal that disruptions in translation elongation trigger a toxic sub-type of transcription–replication collisions, leading to DNA end formation. We narrow our focus to these toxic TRCs driven by disrupted transcription-translational coupling and show that DNA end formation from this TRC sub-type depends on the timing of replication initiation. Specifically, these DNA ends arise from collisions between a stalled replication fork and an upstream daughter fork—referred to here as replication fork rear-ending [18]. Contrary to existing models suggesting DNA end repair through recombination or replication restart [18], the resulting DNA ends are instead resolved by a genome-wide degradation pathway mediated by RecBCD, leading to “replication reset” of the replication cycle.

## Materials and methods

Resources table see Table 1.

**Table 1.** Resources table

Reagent or resource	Source	Identifier
Bacterial and virus strains		
P1*vir*	Lab Stock	N/A
HME45	Lab Stock	N/A
Chemicals, peptides, and recombinant proteins		
Carbenicillin	Thomas Scientific	C46000
Chloramphenicol	Fisher Bioreagents	BP904
Kanamycin A	Sigma–Aldrich	K1377
Tetracycline	Sigma–Aldrich	T3383
Anhydrotetracycline	Chemodex	A0197
Doxycycline	Alfa Aesar	J60422
Rifampicin	TGI	R0079
Cephalexin	Sigma–Aldrich	C4895
FITC	Sigma–Aldrich	46950
DAPI	Sigma–Aldrich	D9542
Rabbit anti-GroEL antibody	Lab Stock	N/A
Rabbit anti-Gam antibody	Lab Stock	N/A
AlexaFluor^®^ 647 goat antirabbit IgG	Thermo Fisher	A-21244
Precision Plus Protein^TM^ Kaleidoscope	Bio-Rad	1610375
Critical commercial assays		
Qiagen FX CDI Kit	Qiagen	180484
Promega Wizard Genomic DNA Purification Kit	Promega	PAA1125
Deposited data		
Short Read Sequencing, see Supplementary Table S7	This study	PRJNA1219134
Experimental models: Organisms/strains		
*Escherichia coli* strains, see Supplementary Tables S5 and S6	This study	N/A
Oligonucleotides		
(Custom NGS Read 1 Primer)5′–GCACGCCCGTCGCTCAGTCCTAGGTATAATACTA–3′	IDT (HPLC Purified)	OC1468
(Custom NGS Index 1 Primer)5′–GATCGGAAGAGCACACGTCTGAACTCCAGTCAC–3′	IDT (HPLC Purified)	OC1469
(Custom NGS Index 2 Primer)5′–TATTATACCTAGGACTGAGCGACGGGCGTGC–3′	IDT (HPLC Purified)	OC1470
[CRISPRi Library Prep polymerase chain reaction (PCR) 1 General Reverse]5′–GTGACTGGAGTTCAGACGTGTGCTCTTCCGATCTAAAGGACCCGTAAAGTGATAATGAT–3′	IDT (Standard Desalting)	OC1332
(CRISPRi Library Prep PCR 1 Forward)5′–TTCCCTACACGACGCTCTTCCGATCTNNNNNNNNGCACGCCCGTCGCTCAGTCCTAGGTATAATACTA–3′NNNNNNNN = i5 Index	IDT (Standard Desalting)	OC1334
(CRISPRi Library Prep PCR 2 General Forward)5′–AATGATACGGCGACCACCGAGATCTACACTCTTTCCCTACACGACGCT–3′	IDT (Standard Desalting)	OC1333
(CRISPRi Library Prep PCR 2 Reverse)5′–CAAGCAGAAGACGGCATACGAGATNNNNNNNNGTGACTGGAGTTCAGACG–3′NNNNNNNN = i7 Index	IDT (Standard Desalting)	OC1335
Recombinant DNA		
EcoWG1 psgRNA Library	Addgene	Pooled Library #131625
pDS596	Dr Jon Kaguni	N/A
pLS120	Dr Jon Kaguni	N/A
pSK762	Lab Stock	N/A
pSK760	Lab Stock	N/A
pUA66-PsulA-GFPmut2	Dr Uri Alon	N/A
Software and algorithms		
Study Github Page	This study	10.5281/zenodo.17088933
R	R Core Team [20]	v4.2.0
ggplot2	Wickham 2016 [21]	v3.4.2
breseq	Deatherage and Barrick 2014 [22]	v0.37.1
FastP	Chen *et al.* 2018 [23]	v0.12.4
Bowtie2	Langmead and Salzberg 2012 [24]	v2.2.5
Deeptools	Ramirez *et al.* 2014 [25]	v3.5.1
Flan	Mazoyer *et al.* 2017 [26]	v1.0

### Method details

#### Bacterial strain building

All bacteria used in this study are derivatives of K-12 sub-strain MG1655. Detailed strain genotypes and lineages are listed in Supplementary Tables S5 and S6. Knockout alleles were sourced from the Keio collection or created using standard recombineering techniques. To generate new knockout alleles, the FRT-flanked kanamycin resistance cassette of pKD13 was amplified with primers containing 40 bp of homology to the target gene. Homology arms were designed to include the start codon of the gene of interest, as well as the last 21 bp of the gene, as done during the generation of the Keio collection [27]. Resulting repair templates were recombineered into strain HME45. Knockout alleles were moved into our MG1655-derived strains using standard P1*vir* transduction [28] onto Luria Broth (LB) plates containing selective antibiotic and 6 mM sodium citrate. Transductants were re-struck on such plates twice, to cure the strain of P1*vir*. The presence of gene knockouts was confirmed via PCR of the target locus. Knockout allele antibiotic resistance markers were removed by transforming with the plasmid pE-Flp, re-streaking resultant colonies at 37°C, and then verifying loss of pE-Flp [29] and antibiotic resistance through an additional re-streak. When working with knockouts with known sensitivity phenotypes, such as ultraviolet light-sensitivity, these phenotypes were confirmed prior to moving forward with experiments. Nonknockout alleles were co-transduced with nearby Tn10 or I-Deconvoluter [30] collection markers. The presence of the correct allele was determined phenotypically and via Sanger sequencing of the point mutation. Strains with suspected suppressor alleles or known mutator phenotypes were whole genome sequenced and presence of a clean strain background was verified with Breseq [22].

For plasmid transformations, saturated LB cultures of cells were serially pelleted and washed with ultrapure water, before transformation via electroporation—1.40 kV voltage, 25 µF capacitance, 200 Ω resistance, and 0.1 cm cuvette gap length.

#### Bacterial culturing

Bacterial liquid culturing was done in a 37°C water bath using LB media (Lennox formulation), unless otherwise indicated. All liquid culturing experiments were done in 500 ml baffled Erlenmeyer flasks at a 50 ml culture volume, 150 rotations per minue (RPM) agitation, and sheltered from ambient light, unless otherwise indicated. Plating was done on 1.5% agar LB plates. When relevant, glucose or arabinose sugars were supplied at final concentrations of 0.2%. Induction of tetracycline-sensitive promoters was accomplished with 200 ng/ml anhydrotetracycline hydrochloride. Selective antibiotics were applied at concentrations of 50 µg/ml for carbenicillin, 12.5 µg/ml for chloramphenicol, 40 µg/ml for kanamycin A, and 12.5 µg/ml for tetracycline. Tetracycline concentrations were halved when sodium citrate was present. Kanamycin A concentrations were halved when working with strains sensitive to, or possibly sensitive to, translation stress. For experiments that indicate growth in Tryptone Broth (TB) or minimal media, formulations are 10 g tryptone, 2.5 g NaCl per liter for TB or MinA salts supplemented with 1 mM MgSO_4_. For experiments where strains were serially diluted, such dilutions were done in MinA salts supplemented with 1 mM MgSO_4_, with no supplied carbon source.

#### Bacterial growth curve profiling

Growth curve experiments were done in a 24-well format, 1 ml final culture volume. Saturated cultures were used to seed the indicated media 1:1000 (∼3 × 10^6^ cells/ml). Well plates were immediately placed in a Cytation 5 plate reader with its growth chamber pre-warmed to 37°C. For 24 h, the cells are shaken intermittently (15 s shake, double orbital pattern, 282 cpm, 3 mm circle radius) and OD_600_ is measured at 15-min intervals. Growth curve data were aggregated and plotted in R using ggplot2 with error bars corresponding to one standard error of the mean (SEM) at each timepoint (Fig. 2A). Quantification of exponential phase growth rate was done by subtracting OD_600_ T_5.5 h_ from OD_600_ T_6.5 h_ (Supplementary Fig. S2E).

#### Measurement of viable colony forming units (CFU) (growth curve)

Saturated cultures of wild-type or Gam-expressing cells were diluted 1:1000 into LB media supplemented with 200 ng/ml anhydrotetracycline and 1.25 µg/ml chloramphenicol. At the indicated timepoints, 1 ml aliquots of the culture were pulled, serially diluted, and plated on LB plates. Viable CFUs were counted after 24 h.

#### Measurement of viable CFU (plate selection)

Saturated cultures of the indicated genotype were serially diluted and plated on TB plates supplemented with glucose and 200 ng/ml doxycycline, TB plates supplemented with glucose and 200 ng/ml anhydrotetracycline, or LB plates supplemented with 40 µg/ml kanamycin A. Due to the instability of doxycycline, care was taken to avoid exposing the plates to light and plating was done within 24 h of pouring the plates. Colonies were counted after 24 h of incubation for doxycycline and anhydrotetracycline experiments and 48 h of incubation for kanamycin A experiments. All viable colony counts were normalized to LB-only titer plates. Where plasmids are used, experimental plates and titers included selection for the plasmid. For each genotype/condition, 8–9 replicates were acquired over 2–3 different experiments. Normalized viability was plotted with error bars corresponding to 95% confidence intervals (CI). Significance was assessed with pairwise *t*-tests (paired = FALSE, exact = TRUE, p.adjust.method = ‘holm’). Significance was represented with *, **, and *** corresponding to *P* <.05, .01, and .001, respectively.

#### Fluctuation assays (spontaneous rifampicin-resistance; AmpD loss of function selection [31]; cI^857^ loss of function selection [32])

Seed cultures for each given genotype were inoculated, from single colonies, in 3 ml of LB media and allowed to saturate overnight at 37°C. Saturated seed cultures were serially diluted 1:10 000 and 100 µl of diluted seed culture per experimental culture were used to seed 10 experimental cultures (∼5 × 10^4^ cells per 3 ml inoculum). For standard fluctuation assays, experimental cultures were inoculated in 3 ml LB media or 3 ml LB media supplemented with 1.25 µg/ml chloramphenicol. These experimental cultures were incubated at 37°C overnight, until saturation. Saturated experimental cultures were titered on LB plates and an aliquot of cells were plated on antibiotic selection: 100 µl of undiluted culture onto LB + 50 µg/ml rifampicin; 1000 µl of undiluted culture onto LB + 100 µg/ml ampicillin; 100 µl of undiluted culture onto LB + 12.5 µg/ml tetracycline. Titer plates were counted after 24 h and antibiotic selection plates were counted after 48 h.

Mutation rate per generation, *µ*, was determined through the Ma–Sandri–Sanker Maximum Likelihood Estimation method using the R package Flan function *mutestim* and statistical significance was assessed using the Flan function *flan.test* with a one-sided alternative.

#### Phase contrast microscopy of exponentially growing cells

Saturated cultures of wild-type or Gam-expressing cells were diluted 1:1000 into LB media supplemented with 200 ng/ml anhydrotetracycline. Flasks were incubated until cells reached an OD_600_ of 0.2–0.4. A 1 ml aliquot of cells was then taken and cell growth was arrested by brief incubation on ice. Cells were then pressed onto glass slides and quickly imaged at 100× magnification on a Zeiss Observer.Z1 microscope equipped with a Photometrics Prime BSI sCMOS camera. At least five images were taken per slide and the experiment was repeated three times.

#### SOS reporter quantification

Saturated cultures, grown in LB supplemented with 20 µg/ml kanamycin A to maintain reporter plasmid were diluted 1:1000 in LB media supplemented with 20 µg/ml kanamycin A and 200 ng/ml anhydrotetracycline. Flasks were incubated until cells reached an OD_600_ of 0.2. When relevant, 8.5 ng/ml ciprofloxacin or 1.25 µg/ml chloramphenicol were then spiked into the culture. After an additional 30 min of growth, aliquots of cells were placed on ice. Chilled cells were pelleted by centrifugation at 17 000 × *g* for 1 min and resuspended in 0.22 µm-filtered phosphate buffered saline (PBS) at a final concentration of ∼10^7^ cells/ml. Diluted cells were analyzed on an Attune Acoustic Focusing Cytometer with event rates of ∼1000–2000 events per second. Per sample, ∼20 000 GFP^+^ events were collected. For visualization of averaged replicates, an equivalent number of post-gating events were combined and graphed in R with ggplot2’s geom_density() function.

#### SOS reporter quantification (bulk measurement)

Saturated cultures, grown in LB supplemented with 20 µg/ml kanamycin A to maintain reporter plasmid were diluted 1:1000 in LB media supplemented with 20 µg/ml kanamycin A and 200 ng/ml anhydrotetracycline. Flasks were incubated until cells reached an OD_600_ of 0.2. When relevant, 8.5 ng/ml ciprofloxacin or 1.25 µg/ml chloramphenicol were then spiked into the culture. After an additional 30 min of growth a 10 ml aliquot of cells was then pelleted at 4000 × *g*, for 10 min, at 4°C, drained, and resuspended in 150 µl of Tris-EDTA (TE) buffer. The concentrated cells were pipetted into a 96-well plate, transferred to a Cytation 5 plate reader, and OD_600_ and GFP fluorescence (Ex: 465 ± 20; Em: 515 ± 20) were quantified. OD_600_ and fluorescence signal were background-subtracted before GFP fluorescence was normalized to OD_600_ reading and all values were further normalized to the average untreated sample signal.

#### Rifampicin-cephalexin run-out quantification of replicating chromosome copy number (done as in [33])

Saturated cultures were diluted 1:1000 in LB media, supplemented with 200 ng/ml anhydrotetracycline or 1.25 µg/ml chloramphenicol, when relevant. All cell collections were done by transferring 3 ml of culture into 7 ml of −20°C 100% ethanol and immediately vortexing at maximum strength for 10 s. Flasks were incubated until cells reached an OD_600_ of 0.2. For quantification of exponential phase chromosome copy number, cells were collected and placed at 4°C until the end of the experiment. Remaining cultures were spiked with 150 µg/ml rifampicin (or 125 µg/ml chloramphenicol for Fig. 3A) and 10 µg/ml cephalexin. Cultures were given 2 h to continue chromosome replication before collection.

Fixed cultures were pelleted at 4000 × *g* for 10 min, washed once in filtered PBS, and resuspended in PBS supplemented with 3 µg/ml fluorescein isothiocyanate (FITC) and 2 µg/ml 4',6-diamidino-2-phenylindole (DAPI). Cells were stained for 30 min at 4°C in the dark. Cells were then diluted to a concentration of 10^7^ fixed cells/ml and analyzed on an Attune Acoustic Focusing Cytometer with event rates of ∼1000–2000 events per second. Per sample, ∼20 000 FITC^+^/DAPI^+^ events were collected. For visualization of averaged replicates, an equivalent number of post-gating events were combined and graphed in R with ggplot2’s geom_density() function.

To estimate peaks in cells chromosome count from unnormalized DAPI signal, the second derivative of the intensity distribution was used to identify points where change in intensity switched from positive to negative. To convert DAPI signal to chromosome count, DAPI signal for peak positions was plotted against assigned control chromosome number and a linear regression equation was modeled on the data, resulting in a DAPI to chromosome conversion.

#### Rifampicin-cephalexin run-out sequencing (Rif-Seq)

Saturated cultures were diluted 1:1000 in LB media, supplemented with 200 ng/ml anhydrotetracycline when Gam induction was desired. Flasks were incubated until cells reached an OD_600_ of 0.4. Cultures were spiked with 150 µg/ml rifampicin and 10 µg/ml cephalexin. These cultures were given 2 h to continue chromosome replication before being incubated on ice and pelleted. Genomic DNA (gDNA) was then isolated using the Promega Wizard gDNA isolation kit and this gDNA was prepared for next-generation sequencing (NGS) using the Qiagen FX CDI library preparation kit with a 1 µg DNA input and a 7.5-min fragmentation time. Samples were sequenced on an Illumina NextSeq 550 platform with paired-end 37 × 37 read length to an average sequencing depth of ∼4M reads per sample. Raw .fastq files were processed with custom bash and R scripts. Briefly, fastq files were quality filtered using FastP and mapped with Bowtie2 to the NC_000913.3 reference genome. Resulting Sam files were converted to Bam format and coverage was computed using Deeptools bamCoverage. The resulting bedgraph files were averaged, smoothed, outlier trimmed, and plotted using a custom R script.

#### CRISPRi-synthetic essentiality screening

All screened strains were built in duplicate and from each duplicate strain, two independent colonies were taken in parallel through the entire process. Strains containing an inducible dead Cas9 construct were transformed with the EcoWG1 psgRNA library [34] to a minimal library coverage of 10^7^ transformants per aliquot of cells. Transformant aliquots were then used to seed a 50 ml LB culture supplemented with 30 µg/ml kanamycin A in a 500 ml baffled Erlenmeyer flask. This culture was grown in a 37°C water bath, at 150 RPM, to an OD_600_ of 0.2–0.4. A timepoint zero aliquot was pulled and plasmid was isolated. Then for each genotype-replicate, three LB 30 µg/ml kanamycin A, 200 ng/ml anhydrotetracycline plates were seeded with 0.01 OD equivalents of exponential phase cells and allowed to grow at 37°C for 24 h. The resulting lawns were then scraped from the plates in 3 ml of MinA 1 mM MgSO_4_ salts, with no carbon source, diluted 1:1000 and 60 µl of cells were used to seed three more LB 30 µg/ml kanamycin A, 200 ng/ml anhydrotetracycline plates. The plates were again grown for 24 h at 37°C and lawns were subsequently scraped. Plasmid was isolated from the resulting cell slurry.

#### CRISPRi-synthetic essentiality library preparation

Library preparation consists of two sequential PCR reactions. First 100 ng of input plasmid is amplified in a 50 µl reaction (Phusion buffer HF, 200 pM dNTPs, 0.5 pM OC1332, 0.5 pM OC1334, or derivative with alternate i5 index, and 1 U Phusion polymerase) for nine cycles (1 × 95°C for 2 min; 9 × 98°C for 20 s, 50°C for 15 s, and 72°C for 30 s; 1 × 72°C for 2 min). Five microliters of this first reaction is then spiked into the second 50 µl PCR reaction (Phusion buffer HF, 200 pM dNTPs, 0.5 pM OC1333, 0.5 pM OC1335 or derivative with alternate i7 index, and 1 U Phusion polymerase) for an additional nine PCR cycles. The final reaction was then cleaned up with a 1× volume of Ampure XP beads. Pooled libraries were then sequenced on an Illumina NextSeq 550 platform with single end 20 bp reads and 8 × 8 dual index read settings. Custom read 1, index 1, and index 2 primers: OC1468, OC1469, and OC1470 were prepared for sequencing according to Illumina kit instructions. And sequencing was done with a custom Illumina recipe that caused the first two cycles to register as dark cycles.

#### CRISPRi-synthetic essentiality data analysis

Sequencing run folders were demultiplexed with bcl2fastq2 with the settings –minimum-trimmed-read-length 19 and –mask-short-adapter-reads 19, due to the small read length. Raw FASTQ files were converted to singe guide RNA (sgRNA) count tables using a python script slightly modified from Cui *et al.* [35]. Count tables were processed using a custom R script derived from Rousset *et al.* [36] that treats sgRNA counts like RNA seq counts. These counts are fit to a negative binomial distribution and fold changes from pre- and post-dCas9 induction are estimated using Deseq2. Significant deviations in gene essentiality, compared to control, were identified by plotting experimental vs. control fold-change values by-gene. A linear regression model is fit to this plot and if removing a gene from the plot significantly increases the fit of the regression model, then it is called as a significant change.

#### Western blotting of gam expression levels

Cell lysates were prepared by diluting saturated cultures 1:100 in LB 0.2% glucose media, supplemented with 0, 100, 200, or 400 ng/ml doxycycline. Diluted cultures were grown at 37°C to an OD_600_ of 0.5 ± 0.1. One hundred microlitres of each sample culture was pelleted, frozen at −80°C to lyse, resuspended in 2× loading buffer with 5% β-mercaptoethanol, and boiled at 100°C. Protein ladder and 10 μl of each lysate was run on a 10% sodium dodecyl sulphate–polyacrylamide gel at 120 V until separated and transferred to a 0.2 μm PVDF membrane using a Trans-Blot Turbo Transfer system. Membranes were cut at the 50 kDa marker and incubated overnight at 4°C in blocking buffer (2% milk in TBS-Tween 0.2%). Primary antibody (in-house rabbit anti-GroEL at 1:10 000 or in-house rabbit anti-Gam at 1:250) was added to blocking buffer and incubated for 24 h at 4°C. Membranes were washed 3× with TBS-Tween 0.2% and incubated with 1:10 000 secondary antibody in TBS-Tween 0.2% for 1 h at room temperature (Thermo Fisher AlexaFluor^®^ 647 goat antirabbit IgG Cat. #A-21244). Images were taken with 100 ms exposure using the Cy5 setting (Azure 400, Azure Biosystems). Blots were quantified with FIJI. Briefly, background intensity was subtracted from the intensity of GroEL and Gam for each sample. Background-subtracted Gam intensity was then normalized to background-subtracted GroEL intensity.

#### Analysis of END-Seq data

Wild-Type exponential phase and stationary phase END-Seq .fastq files from Mei and Fitzgerald (2021) [7] were downloaded from SRA. FASTQ files were deinterlaced, quality score and duplicate read trimmed, aligned to a reference genome with the terminus region masked, and resulting alignments were binned into 1 kb coverage bins, by strand. Data were plotted as Loess-smoothed, normalized coverage in R.

### Quantification and statistical analysis

All quantifications of plating viability consist of 6–9 replicates acquired on at least two different days of experimentation. All data representations that are composites of multiple replicates, such as NGS data, growth curves, and flow cytometry density plots are labeled with their replicate count on their respective figure legends. Additional information on data replication is included in each experiment’s respective ‘Method details’ section.

In all cases where significance is assigned, the following symbols: n.s., *, **, and *** represent *P *>.05, *P* ≤.05, *P *<.01, and *P *<.001, respectively. CIs are labeled in figure legends as either 1 SEM or 95% CI. All tests of significance were done with pair-wise Student’s two-sided *t*-tests, with Holm multiple testing corrections, unless otherwise specified. Plating viability statistics were done with two-sided alternatives. All other statistics were done with one-sided alternatives.

## Results

### Translation stress causes recombination-insensitive DNA ends.

**Figure 1.**
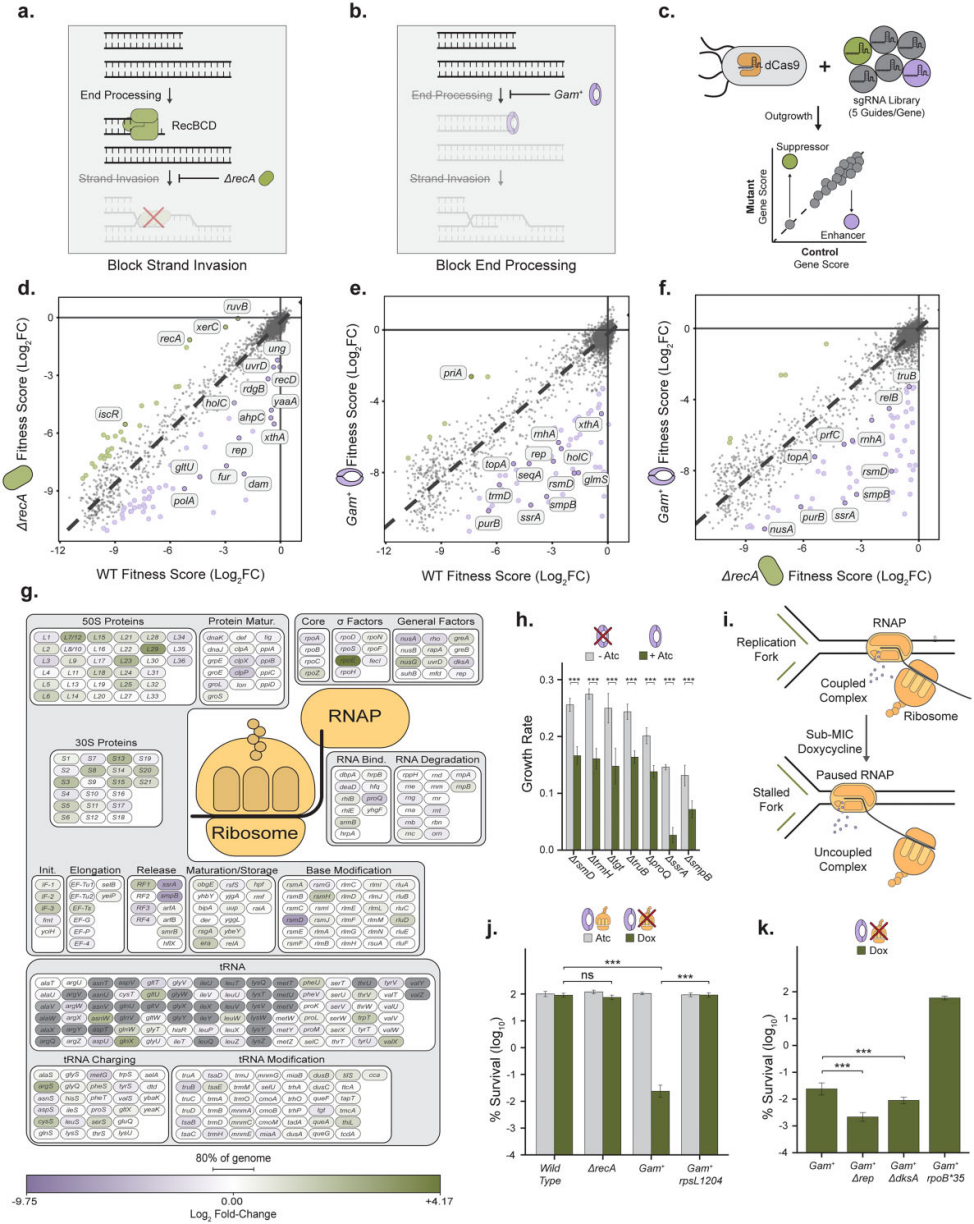
Translation elongation disruptions promote recombination-insensitive DNA end formation via TRCs. (**A**) Absence of the RecA recombinase blocks DNA end repair at the step of DNA stand exchange. (**B**) Gam blocks DNA end processing by RecBCD. (**C**) Output schematic of the CRISPRi-based synthetic interaction screen. Gene knockdowns were assessed for their impact on growth fitness in different backgrounds. Each data point represents relative sgRNA abundance from two experimental conditions. The dashed line represents a linear regression model of all data showing no fitness difference between conditions. Green and purple dots represent sgRNA-dependent knockdowns that cause a gain (green) or loss (purple) of cellular fitness. (**D, E**) CRISPRi synthetic gene interaction profiles comparing the growth fitness effects of gene knockdowns in panel (D). *ΔrecA* (*N* = 4) and wild-type (*N* = 3) cells, (**E**) *gam^+^* (*N* = 4) and wild-type cells (*N* = 3). *ΔrecA* cells show hits in known pathways related to iron regulation, oxidative stress reduction, and DNA replication, while *gam^+^* cells exhibit defects specifically in translation elongation, RNA processing, and transcription–replication related genes. (**F**) Comparison of the profiles from *ΔrecA* (**D**) and *gam^+^* cells (**E**) highlighting limited overlap and revealing translation factors uniquely sensitive to *gam* expression. (**F**) Depiction of color-scaled fitness scores from panel (F) of gene knockdowns in transcription, translation, and RNA processing superpathways. (**H**) Representative translation hits. *gam*-dependent growth rate defect, relative to wild-type, of representative gene knockouts derived from panel (E) (*N* = 6; 95% CI). (**I**) Schematic of translation elongation inhibitor-induced translation–transcription uncoupling and the expected effect of uncoupled transcription-translation complexes on replication fork progression. (**J, K**) *gam*-induced killing in translation-stressed cells. Cell survival with 200 ng/ml anhydrotetracycline or 200 ng/ml doxycycline (*N* = 9, 95% CI). Plating for the streptomycin pseudo-dependent *rpsL1204* was done with 50 μg/ml streptomycin.

*Escherichia coli* relies on homologous recombination machinery for DNA end repair, as this organism lacks a nonhomologous end joining pathway, making DNA end production a lethal, selectable phenotype in mutants defective for homologous recombination. To identify factors that modulate spontaneous DNA end levels, we conducted two genome-wide screens to uncover genetic dependencies in DNA break repair-deficient cells. These screens rely on a CRISPRi knockdown screening method. Briefly, strains harboring an inducible *dCas9* construct are transformed with a library of guide RNA-expressing plasmids. *dCas9* encodes a catalytically-inactive Cas9 which binds to but cannot cleave its targets, which blocks RNA polymerase transcription and results in gene silencing. Each plasmid in this library contains a guide targeting a given gene in the genome, which dCas9 will silence, when induced. Robust silencing of a given gene causes a loss of function phenotype and if this loss of function results in a growth advantage over most other cells, cells harboring plasmids expressing this guide will be enriched at the end of an outgrowth period. Conversely, if this loss of function results in a growth disadvantage over most other cells, cells harboring plasmids expressing this guide will be depleted at the end of an outgrowth period. High-throughput sequencing of plasmid abundances before and after an outgrowth period allows for the effect of a given knockdown to be quantified as a function of guide RNA plasmid relative abundances. The first screen was performed in cells lacking RecA recombinase, which allows pre-homologous recombination DNA end processing by exonucleases, but blocks strand exchange required for homologous recombination (Fig. 1A). The second screen used cells expressing phage Mu’s Gam protein, which binds DNA ends and inhibits their processing by DNA exonucleases—such as RecBCD, RecJ, SbcCD, and various other enzymes capable of DNA end processing—for subsequent DNA repair (Fig. 1B). CRISPRi knockdowns of genes whose activities promote spontaneous DNA damage, should gain a growth advantage in these repair-deficient backgrounds and the corresponding guide RNA plasmids will become enriched over an outgrowth period. CRISPRi knockdowns of genes whose activities safeguard against spontaneous DNA damage should result in a growth disadvantage in these repair-deficient backgrounds and the corresponding guide RNA plasmids will become depleted over an outgrowth period. When the enrichment or depletion of these guide RNA plasmids is compared to their enrichment or depletion in a wild-type control, knockdowns in genes that contribute to spontaneous DNA damage will be relatively enriched. Conversely, knockdowns in genes that safeguard against spontaneous DNA damage will be relatively depleted (Fig. 1C and Supplementary Fig. S1A).

As both screens block DNA end repair, we expected the results of these screens to largely overlap, with shared hits representing knockdowns that produce excess DNA ends. We expected hits unique to *ΔrecA* to be representative of RecA’s other functions such as single-stranded DNA (ssDNA) gap repair and we expected to see little to no hits unique to *gam^+^* cells as all DNA ends subject to Gam binding were expected to be normally processed by *recA*-dependent homologous recombination. However, screen hits showed little overlap (Fig. 1D–F and Supplementary Tables S1–S3). The *ΔrecA* CRISPRi screen recapitulated known *ΔrecA* synthetic interactions [5, 37], with knockdowns of iron regulation (*fur*), oxidative stress reduction (*ahpC, yaaA*), base excision repair (*rdgB, xthA*), and DNA replication (*rep, dam, polA, holC*) genes showing negative synthetic interactions (Fig. 1D, Supplementary Fig. S1B, and Supplementary Table S1).

Synthetic growth defects in both *ΔrecA* and *gam^+^* cells highlight processes that harm genome stability when disrupted, such as R-loop prevention (*rnhA, topA*) [38], genome supercoiling maintenance (*topA*) [39], and nucleotide pools replenishment (*purB*) [40] (Fig. 1D and E, and Supplementary Tables S2G and S3). Comparison of *gam*^+^ and *ΔrecA* phenotypic profiles highlights a strong differential signal (Fig. 1F). Cells expressing *gam* appeared to be hypersensitive to disruption of pathways converging on transcription, transcript processing, and translation (Fig. 1F and G and Supplementary Table S2). We confirmed that knockout of some genes necessary for efficient translation elongation (*rsmD, trmH, tgt, truB*), transcript chaperone activity (*proQ*), and stalled ribosome turnover (*ssrA, smpB*) cause a *gam*-induction specific growth defect (Fig. 1H).

Most *gam*-dependent genetic interactions converge on disrupting translation elongation (Fig. 1F–H). Translation elongation is the major target of multiple antibiotic classes, represented by doxycycline, chloramphenicol, and kanamycin (Fig. 1I). We find that plating of cells on sub-inhibitory doses of doxycycline does not cause lethality to wild-type or *ΔrecA* cells (Fig. 1J), but causes ∼10 000-fold lethality to *gam^+^* cells (Fig. 1J and Supplementary Fig. S2A and B). This *gam*-dependent sensitivity to translation elongation inhibitors extends to other elongation inhibitors, such as chloramphenicol, but cannot be recapitulated by inhibiting translation initiation, with kasugamycin, or other pathways such as cell wall assembly, with carbenicillin (Supplementary Fig. S2A). This effect is not dependent on *gam* dosage effects (Supplementary Figs S2C and S3), as treatment with the nonribosome binding tetracycline derivative, anhydrotetracycline [41], does not induce *gam*-dependent lethality (Fig. 1J and Supplementary Fig. S2D) or cause *gam*-dependent growth defects (Supplementary Fig. S2E and F). Notably, we found that *gam*-dependent translation inhibitor sensitivity was independent of R-loop resolution since DNA end formation was not rescued by induced R-loop resolvase activity (Supplementary Fig. S2H), or Rnase HI overexpression (Supplementary Fig. S2I).

Rather, *gam*-dependent translation inhibitor sensitivity could be rescued by the fast translation elongation phenotype of the *rpsL1204* allele [42] (Fig. 1j). Impaired or slowed ribosome translocation is associated with uncoupling of the ribosome from RNA polymerase [43], pausing or backtracking of the RNA polymerase complex [44, 45], and subsequent TRCs [9]. TRCs cause DNA end formation and are suppressed by the activities of the Rep helicase [11] and RNA polymerase accessory factor, DksA [8, 10]. Removal of either of these factors increased the sensitivity of *gam^+^* cells to doxycycline, suggesting that the observed DNA end formation in response to doxycycline-induced RNA polymerase-ribosome uncoupling (Fig. 1F–H and J) is the result of TRCs (Fig. 1K). Additionally, the sensitivity of *gam^+^* cells to doxycycline is rescued by the rpoB*35 allele (Fig. 1K), an RNA polymerase mutant that destabilizes RNA polymerase on DNA templates [46] and mitigates TRC-induced DNA end formation [9].

The lack of increased sensitivity to translation sub-inhibition in a *ΔrecA* background (Fig. 1D and J), despite clear evidence of TRC-induced DNA end formation in this condition (Fig. 1E–K), suggests that these DNA ends are resolved through a pathway that operates independently of the RecA homologous recombination pathway.

### Collision-induced DNA ends are causes by replication fork rear-ending

**Figure 2.**
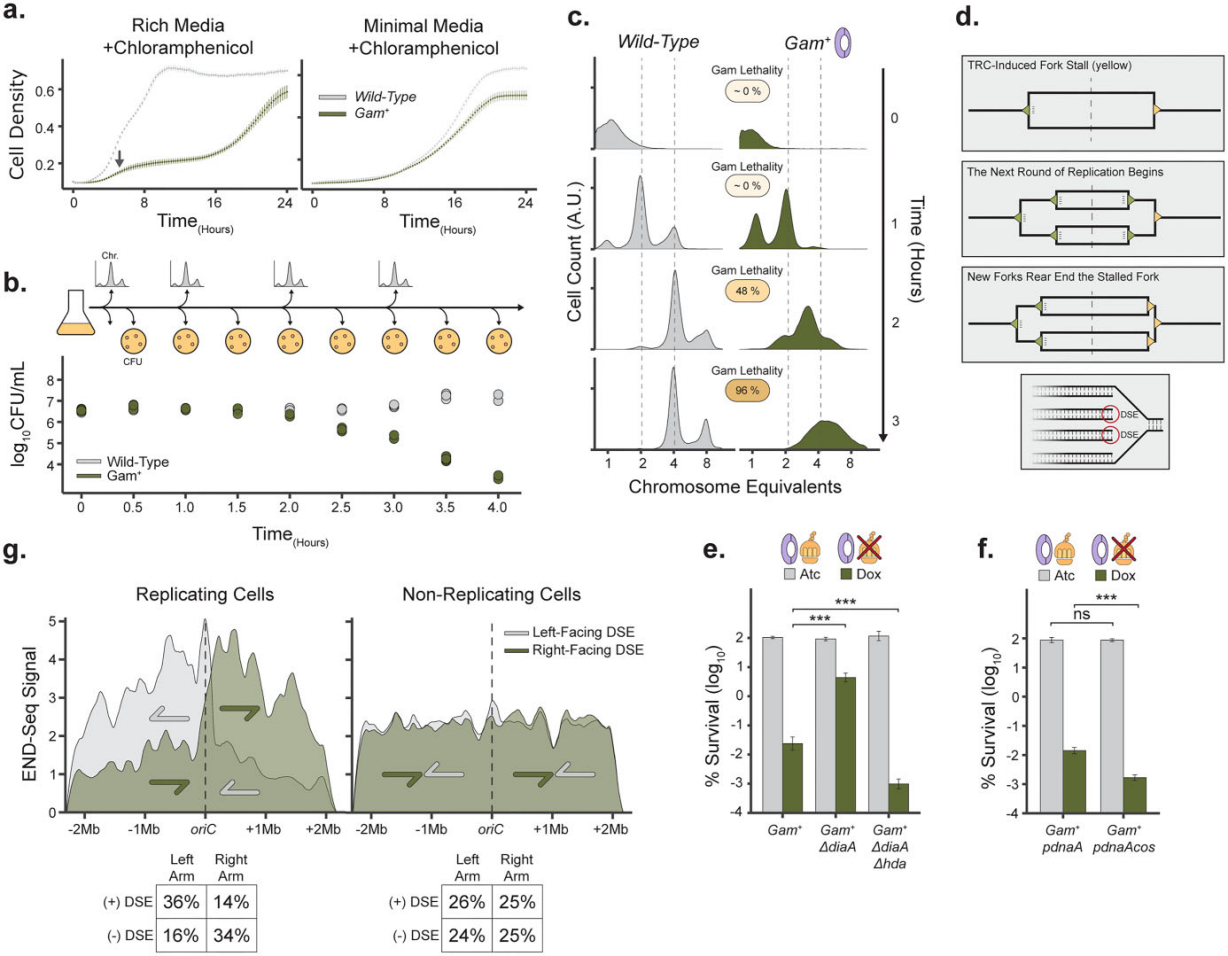
Multiforked replication causes DNA ends through fork rear-ending during translation stress. (**A**) Growth curves of wild-type or *gam^+^* cells grown in fast or slow replicating medium supplemented with a sub-inhibitory dose of translation elongation inhibitor, chloramphenicol (1.25 µg/ml) (*N* = 12; 1 SEM). (**B**) Survival of wild-type and *gam^+^* cells grown in rich media supplemented with 1.25 µg/ml chloramphenicol, as cells transition from stationary phase (T0) to exponential growth (*N* = 4). Samples for DNA replication run-out experiments in Fig. 2C and Supplementary Fig. S5B and C were taken from such cultures. (**C**) Chromosome content of wild-type or *gam^+^* cells, where replication initiation is arrested and existing forks are given time to finish active replication cycles. The indicated time corresponds to time in panel (B). *gam-*lethality refers to CFU relative to timepoint 0 in panel (B). Profiles represent composites of *N* = 2 replicates from Supplementary Fig. S5C. (**D**) Visual representation of TRC-induced replication fork rear-ending model and the rear-ending specific unidirectionality of DNA ends. (**E**) Inter-replication timing contributes to DNA end formation. *gam*-induced killing in translation-stressed DNA replication initiation mutants. Cell survival with 200 ng/ml anhydrotetracycline or 200 ng/ml doxycycline (*N* = 9, 95% CI). (**F**) *gam*-induced killing in translation-stressed DNA replication initiation mutants. Cell survival with 200 ng/ml anhydrotetracycline or 200 ng/ml doxycycline with low level expression of *dnaA* or the dominant pro-initiation mutant *dnaAcos* (*N* = 9, 95% CI). (**G**) END-Seq signals are predominantly one-ended and oriented in the direction of fork progression (left-facing and right-facing signals are shown in light gray and dark green, respectively) in replicating cells. Nonreplicating cells exhibit no such bias (*N* = 2).

The exact mechanism by which TRCs lead to DNA end formation is subject to debate, as multiple mechanisms of DNA end formation have been proposed. In one proposed mechanism, TRCs cause incorporation of RNA into nascent DNA, resulting in RNase-dependent ssDNA gaps which can be converted to double-stranded DNA (dsDNA) breaks by subsequent replication [9]. Another prominent mechanism is replication fork regression, where nascent strands of a replication fork structure anneal, forming a four-way dsDNA junction with a single DNA end [47]. Another mechanism that has been suggested is cleavage of stalled fork structures by holliday junction resolvases, such as RuvC [48]. And finally, replication forks can “rear end” other replication forks that have been stalled, producing two one-ended DNA ends [49]. Each of these mechanisms are possible causes of DNA end formation and likely can be attributed to different replisome contexts or types of TRCs. As the DNA end formation mechanisms for translation stress-induced TRCs remain unclear, we sought to identify how these TRC-dependent DNA ends are being formed, to then identify their repair mechanism. We found that TRC-induced DNA ends are linked to growth rate as *gam^+^* cells treated with a sub-inhibitory chloramphenicol dose exhibited significant growth defects exclusively in rich media during exponential growth (Fig. 2A and Supplementary Fig. S4). This correlates with the major difference in replicative behavior between rich and minimal media: in rich media, cells queue multiple rounds of DNA replication simultaneously, a phenomenon known as multiforked replication (Supplementary Fig. S5A) [50, 51]. In multiforked replication, the first round of DNA replication is initiated, which is sufficient to increase chromosome copy number from one to two and permit a cell division. After a brief period of origin of replication dormancy, another DNA replication initiation event occurs. This event is sufficient to increase chromosome copy number from two to four and will permit a subsequent round of cell division for both daughter cells. The duration of origin of replication dormancy is dependent on the growth rate of the cell—and by extension, media richness [50]. The richer the growth media, the faster the growth, the smaller a period of origin dormancy, and the less time that the first replisome has to create distance between itself and the replisome from the subsequent replication initiation event.

To determine whether DNA end formation depends on the increase in replication initiation rate in rich media, we took stationary-phase wild-type and *gam^+^* cultures which contain on average, a single chromosome per cell and grew them in rich medium supplemented with a sub-inhibitory concentration of chloramphenicol. At various growth time points, we removed the antibiotic by washing cell aliquots and assessed cell viability by plating them on drug-free media. As wild-type cells entered exponential growth, we observed a sharp decline in the viability of *gam^+^* cells (Fig. 2B). We sought to determine whether the onset of *gam^+^* cell inviability was the result of the replication state of the cells at this time point. To determine the replication state of cells at various time points (Fig. 2C and Supplementary Fig. S5A–C), we performed replication fork run-out measurements. This approach blocks cell division and the initiation of new replication rounds while allowing active forks to complete their rounds of chromosome replication. This enables us to measure the ability of cells to finish replication and to assess final chromosome counts per cell by flow cytometry (Supplementary Fig. S5A). Early log-phase cells initially displayed a nonreplicating state, containing a single chromosome per cell (Fig. 2C, 0 h). Within the first hour of transitioning to rich media, these cells initiated the first round of replication, resulting in two chromosomes per cell (Fig. 2C, 1 h). Despite actively replicating, single-forked replicating *gam^+^* cells show no decrease in viability after 1 h (Fig. 2C). At the 2- and 3-h timepoints, both wild-type and *gam^+^* cells transitioned to multiforked replication (Fig. 2C). The onset of multiforked DNA replication in *gam^+^* cells correlates with a loss of cell viability (Fig. 2C, 2 h). At this timepoint *gam*-mediated blockage of DNA end processing resulted in noninteger chromosome counts suggesting that *gam* expression stabilizes previously transient replication intermediates in multiforked cells (Fig. 2C, 2 h). This *gam*-dependent arrest of replication intermediates in multiforked replicating cells is dependent on chloramphenicol (Supplementary Fig. S5B and C) and suggest that the observed DNA end replication intermediates are dependent on uncoupling of the ribosome from RNA polymerase and therefore are TRCs. This suggests that TRCs induce DNA ends through replication fork–fork interactions.

Fork–fork interaction-dependent DNA ends are consistent with a replication fork rear-ending mechanism—a process sometimes referred to as head-to-tail collision [18]. The requirement for this DNA damage mechanism is that one replication fork stalls for long enough that a subsequent replisome has time to catch up to the stalled fork. In turn, this would suggest that transcription–replication collisions cause pausing of the replication fork, allowing subsequent forks to catch up and collide with the stalled fork (Fig. 2D). If this model is correct, the incoming fork rear-ends the stalled fork, producing single-ended DNA ends rather than two-ended breaks in the genome (Fig. 2D). This mechanism implies that DNA end formation depends on the inter-replication time, which buffers replication forks from colliding. Furthermore, the resulting DNA ends should be one-ended, lacking a counterpart on the opposing DNA strand. We find that increasing inter-replication time by removing DiaA, a factor preventing excessive replication initiation, partially rescues *gam^+^* doxycycline-sensitivity (Fig. 2C). Conversely, decreasing inter-replication time through a complementary *Δhda* mutation, which counteracts the function of DiaA, restores *gam^+^* doxycycline-sensitivity (Fig. 2E) and decreasing inter-replication time through expression of the gain of function *dnaAcos* allele, which upregulates replication initiation, also increases *gam^+^* doxycycline-sensitivity (Fig. 2F).

We identified the “endedness” of replicative DNA ends in rapidly replicating and nonreplicating cells from END-seq data generated by Mei and Fitzgerald *et al.* (2021) [7]. We find that most replication-dependent DNA ends are one-ended, with the ends facing away from the replication origin (Fig. 2G) as expected from fork–fork collisions. This bias of one-ended DNA ends observed in growing cells is absent in nonreplicating cells (Fig. 2G).

These results show that TRCs induce DNA end formation by DNA replication fork rear-ending (Fig. 2D). This process generates DNA ends without breaking the parental genome. These DNA ends are not repaired by RecA homologous recombination pathway. However, the clearance of these free DNA ends is essential for cell survival, as blocking DNA end processing with Gam during TRCs is lethal.

### Collision-induced DNA ends trigger chromosome degradation

**Figure 3.**
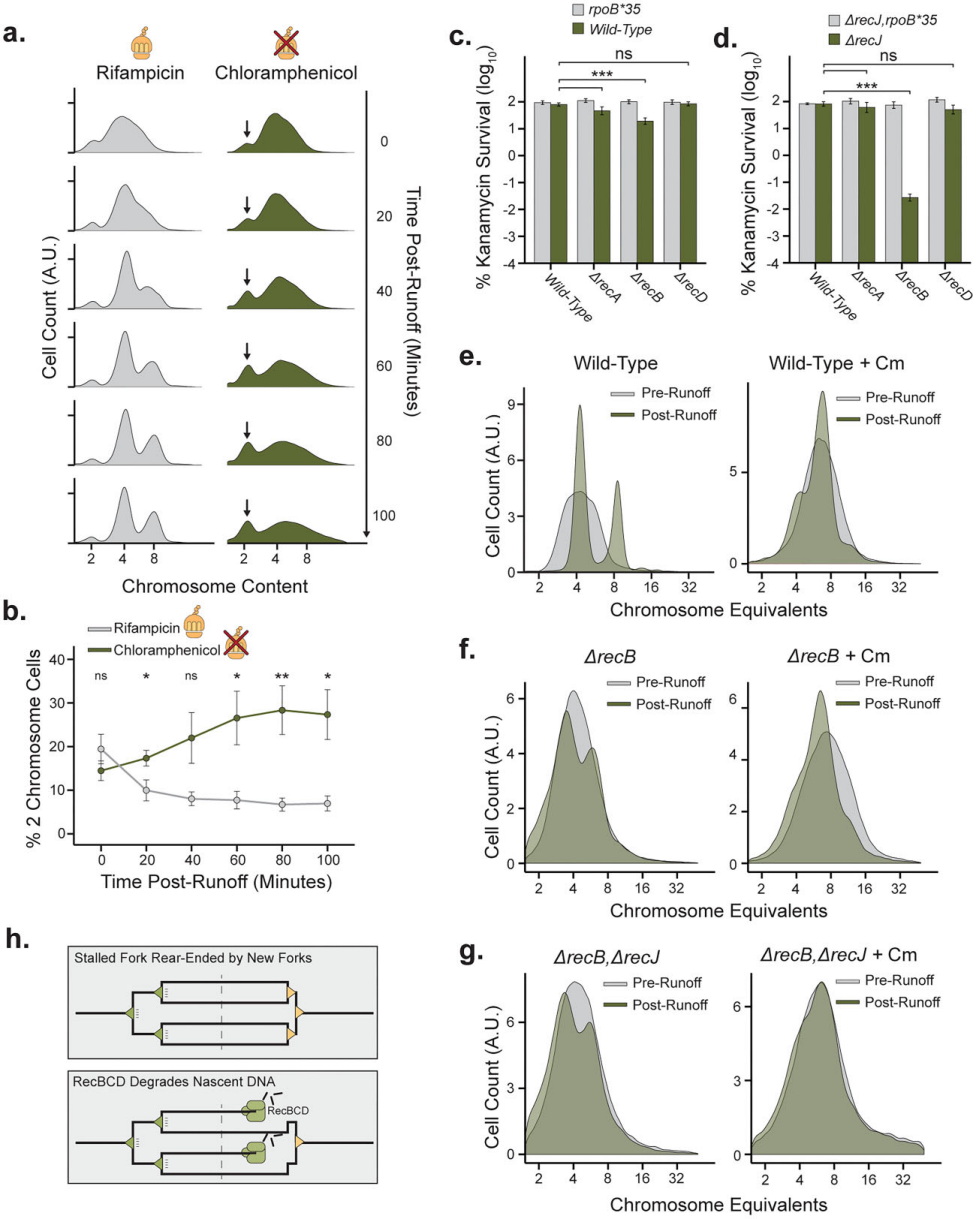
Collision-induced DNA ends trigger chromosome degradation by RecBCD/RecJ exonucleases. (**A**) Replication run-out profiles of chromosome replication state as a function of time after the initiation of run-out (right axis) and drug, 150 µg/ml rifampicin or 125 µg/ml chloramphenicol, used to initiate run-out (top label). DAPI staining quantifies DNA content. Profiles represent a composite of *N* = 4 replicates. Arrow indicates growing two chromosome peak. (**B**) Quantification of cells with two complete chromosomes from panel (A), as a fraction of total number of cells (*N* = 4; 1 SEM). (**C, D**) Percent viable cells of the indicated genotype containing a kanamycin resistance cassette when plated on high-dose kanamycin (40 µg/ml) (*N* = 8, 95% CI). (**E–G**) Rifampicin-induced replication run-out profiles of the indicated genotypes growing in LB medium or LB 1.25 µg/ml chloramphenicol. Profiles represent the composite of *N* = 3 replicates after a 120-min rifampicin run-out. (**H**) Schematic of replication fork rear-ending-derived DNA ends being degraded by the RecBCD nuclease.

The apparent lack of TRC-induced homologous recombination (Fig. 1) in the presence of fork rear-ending induced DNA ends (Fig. 2) prompted us to measure the chromosomal DNA content when cells are exposed to TRCs. We performed replication fork run-out experiments in the absence of transcription elongation (high dose rifampicin treated) and in the presence of high levels of TRCs (high dose chloramphenicol treated), sampling chromosome content during replication run-out (Fig. 3A). Both rifampicin and chloramphenicol are known to inhibit replication initiation [52], allowing us to compare the replication run-out in either absence of transcription or translation. In the rifampicin run-out, single- (final two chromosomes), and multiforked (final >2 chromosomes) replicating cells completed replication into full chromosomes after 40–60 min of replication run-out. In the chloramphenicol run-out, single-forked replicating cells completed replication after 40–60 min; however, multiforked replicating cells could not complete replication within 100 min (Fig. 3A). Notably, as these run-out experiments are derived from the same split cultures, the TRC-free rifampicin run-outs had ∼10% of the population finish the run-out with two chromosomes (Fig. 3B). Conversely, the chloramphenicol-induced TRC run-outs gradually increased in the number of two chromosome cells, indicating that the multiforked replicating cells were reverting to a pre-multiforked state (Fig. 3B). This reversion is the result of cells degrading DNA present at the beginning of the run-out experiment. During these experiments, cell division is inhibited by the cephalosporin, cephalexin, meaning that decreases in DNA content per cell cannot be attributed to additional cell division, and rather, can only be explained by degradation of dsDNA.

To identify the factors necessary for the large-scale DNA degradation seen under TRC conditions, we designed a screen that exacerbates TRCs in the presence of chloramphenicol. Our initial DNA end-sensitivity assays confirmed that the Rep helicase helps clear roadblocks on the DNA, thus preventing DNA end formation (Fig. 1D, E, and K, and Supplementary Fig. S6A) [11]. Accordingly, we carried out a CRISPRi screen in cells lacking Rep, both with and without chloramphenicol, and found that RecBC and RuvAB are major contributors to coping with these fork collisions (Supplementary Fig. S6B and C). The RecBCD complex is the principal exonuclease in *E. coli*, and this screen of *Δrep* growth in sub-MIC chloramphenicol indicates that it is the most likely driver of the observed DNA degradation during TRCs. Consistent with this observation, we found that *∆recB::Kan^R^* cells, but not *∆recA::Kan^R^* exhibited sensitivity to high doses of kanamycin, despite their antibiotic resistance markers (Supplementary Fig. S7A and B). *ΔrecB* cells show some degree of lethality (Fig. 3C), which is greatly exacerbated by removing the RecJ exonuclease, a factor that can partially compensate for the loss of RecBCD activity (Fig. 3D). This lethality is completely rescued by the rpoB*35 allele, which alleviates TRCs (Fig. 3C and D). Finally, compared to wild-type replication run-out profiles (Fig. 3E), replication run-outs of *ΔrecB* cells show large levels of unresolved replication intermediates (Fig. 3F), which is exaggerated by growth in sub-inhibitory doses of chloramphenicol (Fig. 3F). These effects are further exacerbated by co-deletion of *recJ* (Fig. 3F and G), with cells lacking both DNA exonucleases showing a total arrest of TRC-induced DNA degradation over the course of the replication run-out period (Fig. 3G). While cells encountering TRCs do not appear to require homologous recombination for survival, our data show that DNA end processing is dependent on RecBC or accessory RecJ, exonuclease activities (Fig. 3H).

### Chromosome degradation resets an entire replication cycle

To investigate the fate of DNA ends generated by transcription–replication collisions (TRCs) at the DNA level, we employed next-generation sequencing to monitor changes in DNA content across the genome. TRCs under sub-inhibitory translation conditions are not expected to result in locus-dependent degradation. Instead, the degradation effect at the population level is likely to be diffuse across the chromosome, producing a signal that is difficult to interpret without accounting for local decreases in replication elongation [53]. To address this, we utilized a locus-specific TRC to enable unambiguous assignment of causality between TRCs and the resulting DNA degradation.

**Figure 4.**
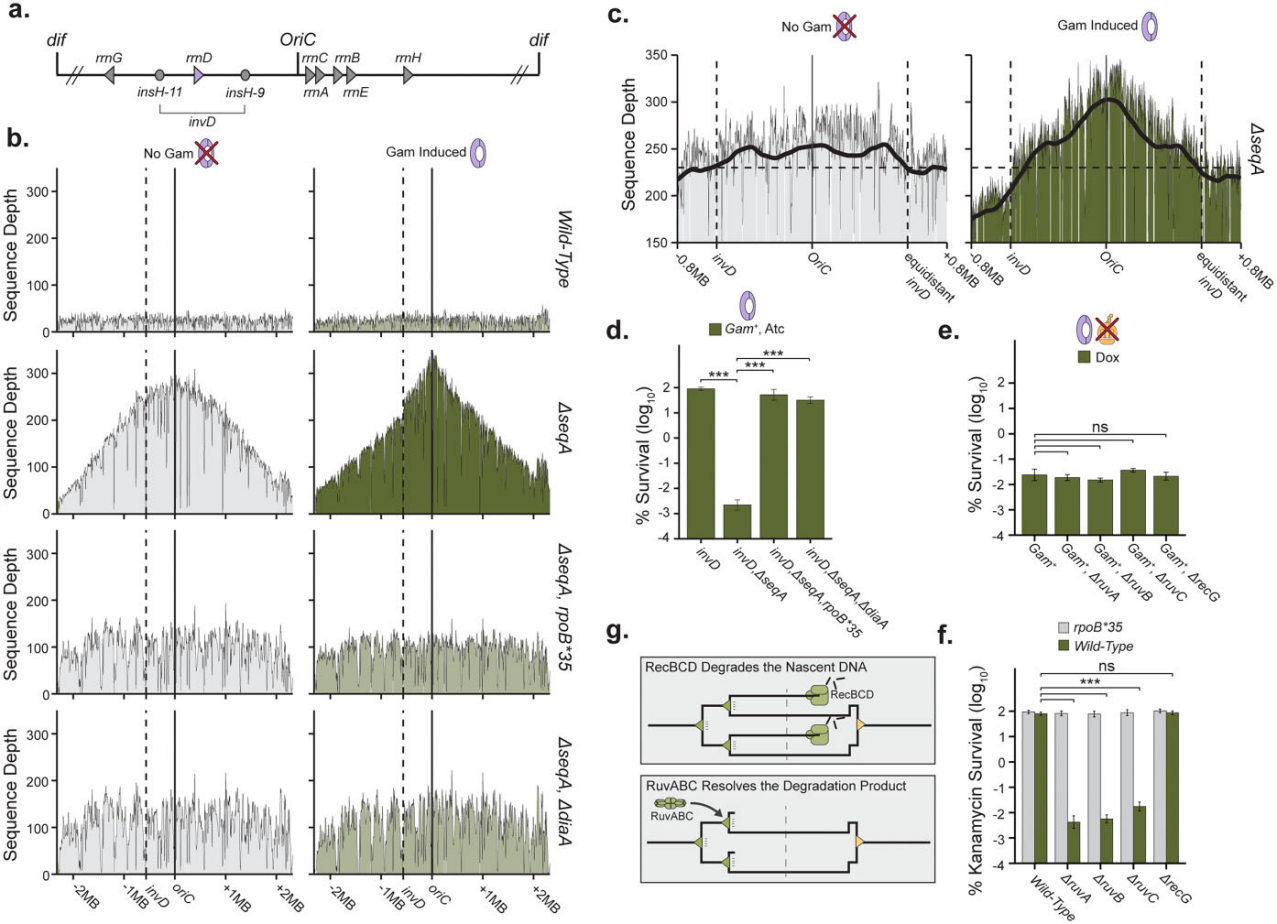
The inverted *rrnD* gene promotes localized TRCs, enabling genomic assessment of replication resets. (**A**) Schematic of chromosome rearrangement in the *rrnD* inversion, *invD. invD* allows a targeted study of TRC-induced stalling and DNA end formation. (**B**) Post replication run-out DNA sequencing profiles for the indicated genotypes (right axis) with and without *gam* induction, reveal DNA degradation originating at the *invD* locus. Dashed line indicates the location of the *invD* locus. Sequencing depth represents counts per million, corrected for terminus copy number. Profiles represent a composite of *N* = 3 replicates. (**C**) Zoomed *ΔseqA* profiles from panel (B). Vertical dashed lines label *invD* locus as well as the locus on the sister replicore equidistant to *invD* from the origin of replication. Horizontal dashed line is for visual comparison between left and right replicores. Gray background line represents Loess-smoothed sequencing depth. (**D**) Percent viable *gam^+^* cells of the indicated additional genotypes, relative to titer (*N* = 9; 95% CI). (**E**) Percent viable *gam^+^* cells plated on 200 ng/ml doxycycline (*N* = 9; 95% CI). (**F**) Percent viable cells of the indicated genotype containing a kanamycin resistance cassette when plated on high-dose kanamycin (40 µg/ml) (*N* = 8, 95% CI). (**G**) Chromosome degradation resets an entire replication cycle in response to replication fork rear-ending.

Previous studies have investigated TRCs by inverting highly transcribed ribosomal operons, reorienting ribosomal RNA (rRNA) transcription to occur head-on with DNA replication at a single locus [54]. We used an inversion of *rrnD*, named *invD*, which allowed us to monitor DNA content at this locus during TRCs during replication fork run-out (Fig. 4A). In an otherwise wild-type background, *invD* does not alter the cell’s ability to complete DNA replication, even if DNA end degradation is blocked by the expression of *gam* (Fig. 4B). This is reflected in an equal sequencing depth across the chromosome. Conversely, in *invD, ΔseqA* cells, we see a delayed completion of the replication cycle, as replication intermediates are still evenly distributed across the chromosome, forming the characteristic DNA pyramid distribution seen in sequenced replicating DNA samples [55]. However, we do not see a complete replicating chromosome pyramid peaking at the origin of replication, rather, it appears that a subset of replication forks were able to bypass the *invD* barrier (represented by the *invD* distal chromosome slope) and a subset of forks were unable to bypass the *invD* barrier, forming a sequencing depth plateau near the replication origin (Fig. 4B and C). Blocking DNA degradation with *gam* expression removes this plateau, resulting in the stabilization of a large number of replication intermediates (Fig. 4B and C). This *gam*-dependent increase in origin-proximal DNA indicates that this DNA is removed from cells when dsDNA exonuclease activity is not inhibited and the plateau is the result of large-scale DNA degradation. This DNA degradation appears to initiate from near the *invD* blockage and extend across the origin of replication to the right replicore sister replisome (Fig. 4C). This degradation pattern suggests that RecBCD degrades well beyond the *invD* locus, extending across the entire nascent chromosome to reset an entire DNA replication cycle. We observe that this large-scale DNA degradation depends on inter-replication time, as the *ΔdiaA* mutation rescues this *ΔseqA, invD* degradation phenotype, and depends on transcription collisions as rpoB*35 similarly alleviates the *ΔseqA, invD* degradation phenotype (Fig. 4B). These phenotypic dependencies are reflected in *gam*-dependent lethality measures with these mutants (Fig. 4D). Further, while the activities of the DNA branch migration, RuvAB, and junction resolution, RuvC, machinery are not required for DNA end formation (Figs 1E and 4E), they are required for endpoint survival in the presence of kanamycin (Fig. 4F) and for *Δrep* fitness when grown in sub-inhibitory chloramphenicol (Supplementary Fig. S6B and C, and Supplementary Table S4). This places the activities of RuvABC after RecBCD degradation (rather than before), likely at the sister replisome [48], rather than in the formation of the DNA end, as suggested in the replication fork regression model. Together, these results suggest a TRC-induced DNA end-specific chromosome degradation pathway, leading to replication reset (Fig. 4G).

### Replication reset by RecBC is nonmutagenic

**Figure 5.**
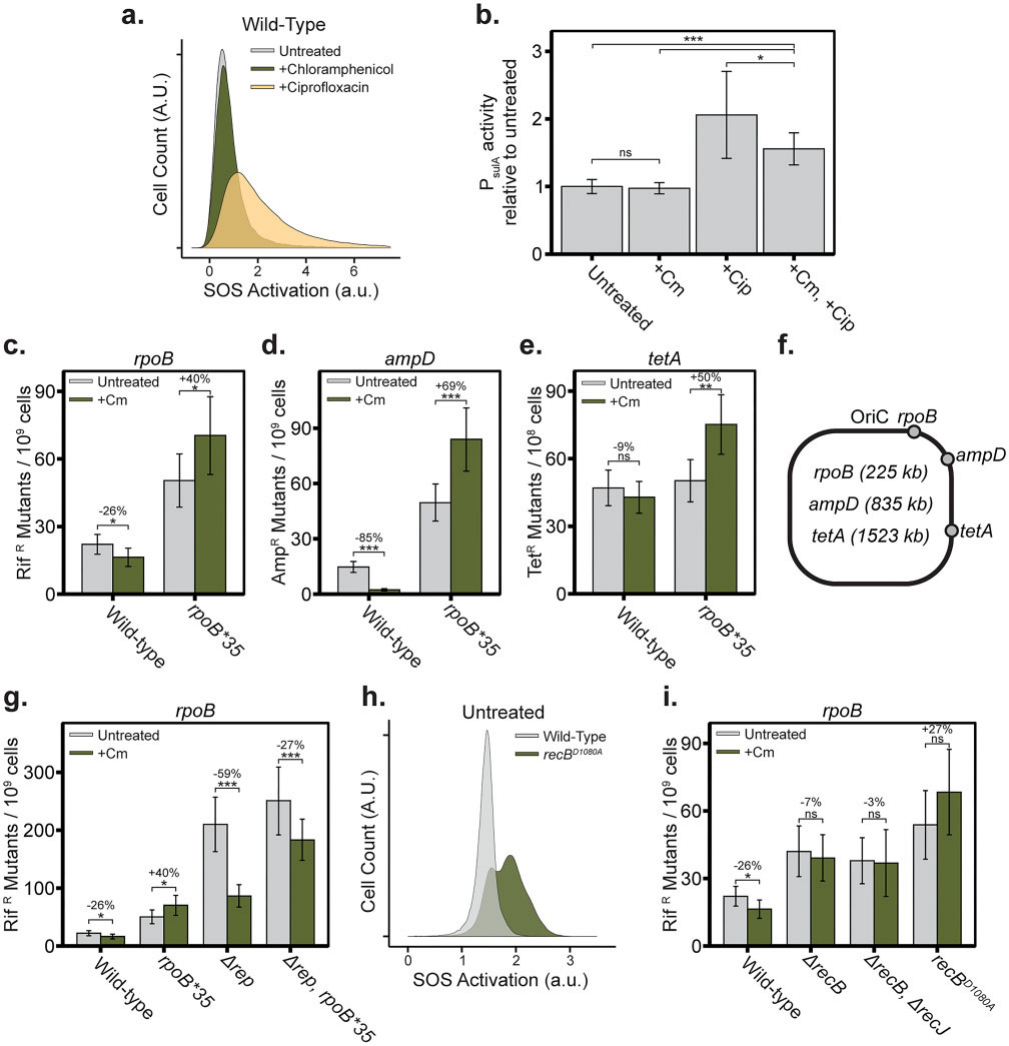
Replication reset does not activate the SOS response or mutagenesis. (**A**) Flow cytometry of *PsulA-GFPmut2* SOS response reporter in exponential cultures untreated, treated with 1.25 µg/ml chloramphenicol, or treated with 8.5 ng/ml ciprofloxacin for 30 min (*N* = 3). (**B**) Bulk measurements of the *P_sulA_-GFPmut2* SOS response reporter done as in panel (A); bulk fluorescent reporter measurements (*N* = 5). (**C–E**) Maximum likelihood estimations of mutation rate per generation using *rpoB* rifampicin resistance mutations [panel (C)], *ampRC*-detection of spontaneous loss of function *ampD* mutations (d), or *cI(ind-)* repressor of *tetA* spontaneous loss of function mutations [panel (e)] as mutation reporters for the given genotypes grown in LB media without (Untreated) or with (+Cm) 1.25 µg/ml chloramphenicol. Error bars represent 95% CIs (*N* = 30). (**F**) Location of the three mutation reporters, relative to the origin of replication (distance in kilobases). (**G**) Maximum likelihood estimations of mutation rate per generation using *rpoB* rifampicin resistance mutations for the given genotypes grown in LB media without (Untreated) or with (+Cm) 1.25 µg/ml chloramphenicol (*N* = 30). (**H**) Flow cytometry of *PsulA-GFPmut2* SOS reporter on wild-type and RecB nuclease null mutant *recB^D1080A^* (*N* = 3). (**I**) Maximum likelihood estimations of mutation rate per generation using *rpoB* rifampicin resistance mutations for the given genotypes grown in LB media without (Untreated) or with (+Cm) 1.25 µg/ml chloramphenicol (*N* = 30).

This mechanism of TRC resolution resets replication through massive DNA degradation up to the sister replisome (Fig. 4B and C), passing multiple chi sites, which should promote recombination and induce the DNA damage response. As observed earlier, replication reset by degradation (Fig. 3A) does not require homologous recombination (Fig. 1F and J). To assess whether TRCs trigger a DNA damage response, we used cells harboring a fluorescent DNA damage reporter, *P_sulA_-GFPmut2*, and treated them with a sub-inhibitory dose of chloramphenicol. Unlike ciprofloxacin-treated cells, which showed a clear reporter induction, the chloramphenicol-treated cells exhibited no such activation (Fig. 5A). To test whether the sub-inhibitory dose of chloramphenicol was capable of masking SOS response activation altogether, we tested whether sub-inhibitory chloramphenicol could block SOS induction by ciprofloxacin. We find that chloramphenicol mutes SOS response induction but does not abolish it, suggesting that the lack of SOS induction during chloramphenicol-only treatment reflects a genuine lack of SOS induction (Fig. 5B). These observations suggest that replication fork collisions are not repaired through a mutagenic homologous recombination pathway and may not be linked to the typical mutagenesis associated with the DNA damage response [56].

To investigate whether chloramphenicol-induced DNA ends cause an increase in mutation rate, we measured mutation rate per generation using three different reporters of spontaneous mutation: *rpoB* rifampicin resistance mutations (Fig. 5C), *ampD* loss of function mutations (which result in *ampRC-*mediated ampicillin resistance) [31] (Fig. 5D), and *cI(ind-)* loss of function mutations (which result in derepression of a tetracycline resistance gene, *tetA*) [32] (Fig. 5E). These mutation reporters are at multiple locations in the chromosome, with *rpoB* and *ampD* being origin proximal and *cI(ind-)* reporter being located towards the terminus-proximal end of the replicore (Fig. 5F). None of these reporters show a chloramphenicol-dependent increase in spontaneous mutation rate (Fig. 5C–E). Rather, the *rpoB* and *ampD* reporters show a significant decrease in mutation rate during growth in chloramphenicol and the *cI(ind-)* reporter shows no change. This trend is reversed in a strain background containing the rpoB*35 allele, which does not show an appreciable rate of TRC-induced DNA ends (Fig. 1K), which shows a significant, chloramphenicol-dependent increase in spontaneous mutation across all three reporters (Fig. 5C–E). This result suggests that sub-lethal chloramphenicol treatment actually functions to lower the spontaneous mutation rate and that this effect is likely dependent on DNA end formation. If so, then exacerbating the effect of chloramphenicol-stalled transcription complexes on DNA end formation should amplify the magnitude of this decrease in mutation rate. To do so, we repeated the *rpoB* mutation reporter assays in a *Δrep* background, which is impaired in its ability to clear transcription complexes which obstruct the replisome. Here, we see that the chloramphenicol-dependent decrease in spontaneous mutation amplifies more than two-fold, from 26% in wild-type cells to 59% in *Δrep* cells (Fig. 5G). Further, deletion of *rep* causes an intermediate phenotype in an rpoB*35 background, no longer resulting in a chloramphenicol dependent increase in mutation rate (Fig. 5C), but not resulting in the same amplitude of chloramphenicol-dependent decrease in mutation rate seen in *Δrep* cells (Fig. 5G).

Since TRCs are resolved by RecBCD’s exonuclease activity, we investigated the consequence of removing this activity on mutagenesis and on SOS activation during normal growth. In the *recB^D1080A^* mutant, which lacks RecBCD nuclease activity but retains helicase function [57], we observed constitutive SOS activation (Fig. 5H) along with an elevated mutation rate (Fig. 5I) compared to wild-type cells. Further, compared to a chloramphenicol-dependent decrease in mutation rate seen in wild-type cells, *recB^D1080A^* cells have a mutation rate that trends towards a mutation rate increase. This pattern is different than that seen when simply abolishing dsDNA exonuclease activity with *ΔrecB* and *ΔrecB, ΔrecJ* mutations. In these deletion mutants, we do not observe a chloramphenicol-dependent increase or decrease in mutation rate (Fig. 5I). This lack of mutation phenotype is likely because these mutants experience inviability when exposed to translation inhibitors (Fig. 3C and D), implying there is no mutagenic alternative to dsDNA degradation, unless a helicase like *recB^D1080A^* is present. These findings indicate that RecBCD’s nuclease function is essential for the anti-SOS and antimutagenic effects of replication reset.

## Discussion

**Figure 6.**
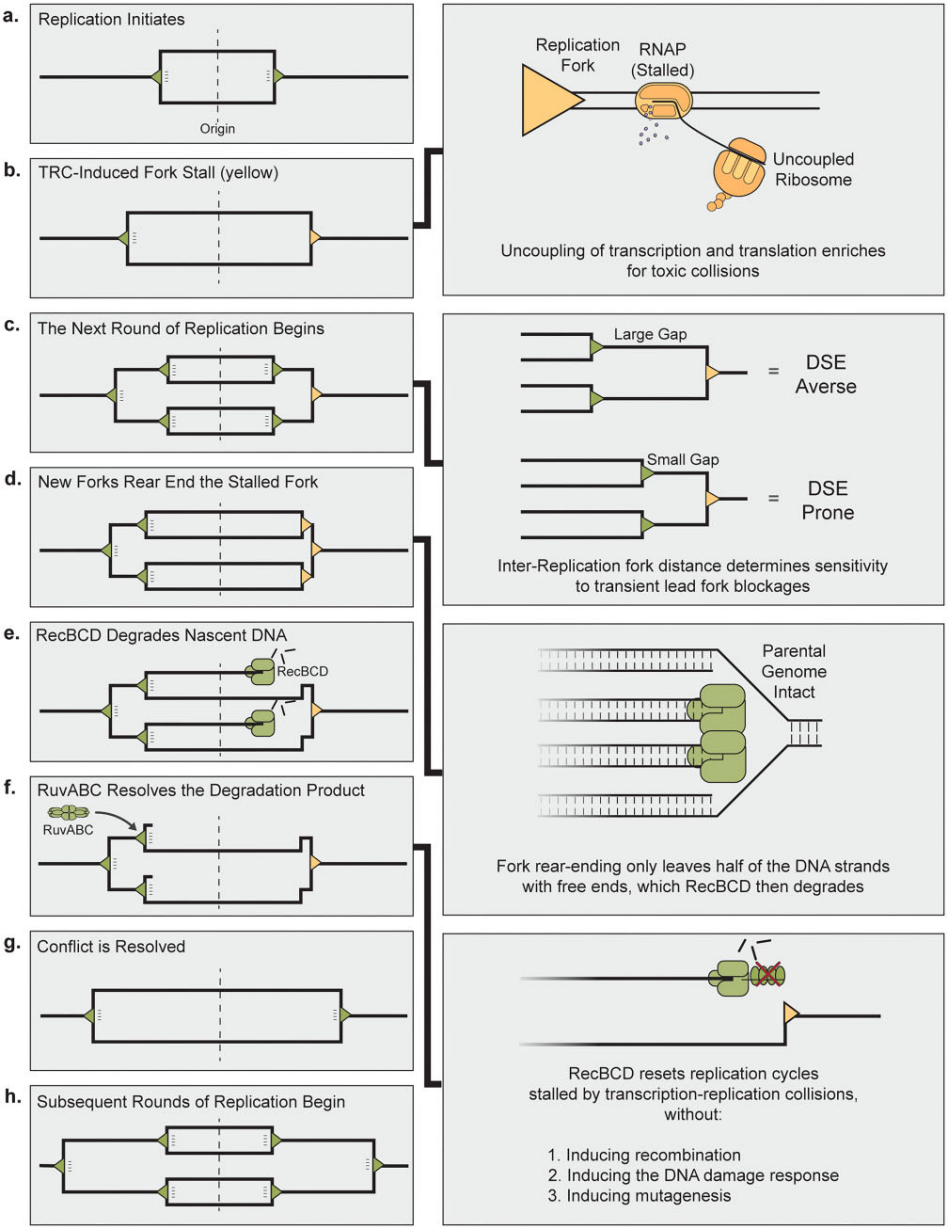
Replication reset drives genome protection. (**A**) An initial round of DNA replication initiates with two replication forks (green triangles). (**B**) At least one replication fork encounters a replication barrier and stalls (yellow). Right box: Uncoupling of transcription and translation causes stalled DNA replication forks. (**C**) Multiforked DNA replication begins with a subsequent round of replication (inner forks). Right box: A large inter-replication initiation time results in a large physical and temporal gap between replication forks. A short inter-replication initiation time results in a small physical and temporal gap between replication forks. (**D**) If the stalled fork remains unresolved, subsequent replication forks rear-end the stalled fork (yellow). Right box: Rear-ending creates DNA ends oriented away from the origin of replication and without chromosome breakage. These ends are substrate for RecBCD binding. (**E**) RecBCD (green) degrades the two fully nascent DNA strands to the other side of the chromosome. (**F**) RecBCD degradation effectively resets a single round of DNA replication. Right box: An unknown factor blocks RecBCD from initiating homologous recombination, while allowing the complex to processively degrade DNA. (**G**) The fork blockage is cleared (both forks now green). (**H**) When subsequent rounds of DNA replication are initiated, the cell is left with a large chromosome buffer, to prevent future DNA damage.

Here, we show that generation of free DNA ends due to translation–transcription uncoupling-dependent TRCs are directly controlled by DNA replication initiation rate and are resolved by large scale DNA resection by RecBCD, independently of its recombination function. We present a model for TRC-induced DNA end formation (Fig. 6A–D), where the primary requirement is a replication fork stalling event (Fig. 6B) caused by transcription stalling due to ribosome uncoupling, followed by subsequent daughter forks rear-ending the stalled fork (Fig. 6C and D). This collapse mechanism does not result in the parental chromosome ever being broken, nor does it require the fork structure to actively dissociate and recruit additional enzymes. However, it leaves open the question of why recombination is not initiated (Fig. 6E and F).

These findings align with the established link between transcription–translation coupling [15] and genome stability. Although earlier work suggested that uncoupling toxicity stems from derepression of ancient foreign DNA [58], eliminating these DNA loci does not fully alleviate the growth defects caused by deleting coupling factors, like NusG, or experimentally uncoupling translation and transcription [59]. Instead, our data show that uncoupling primarily induces replication stress. This uncoupling-induced replication stress is thought to be the result of RNA polymerase entering paused or backtracked states, known to promote replication collisions [9]. Normally, active translation rescues stalled RNA polymerase [17], but when uncoupled, these stalled RNA polymerase are likely resistant to mechanical force that the replisome may use for RNA polymerase removal [60]. In organisms with uncoupled transcription and translation, like *Bacillus subtilis* [61] RNA polymerase is less susceptible to intrinsic pausing signals [62], a possible prerequisite to forming stalled conformations. Together, these observations highlight the diverse evolutionary strategies bacteria employ to prevent RNA polymerase stalling and its detrimental impact on genome stability.

This replication reset model suggests that a replication fork can remain stalled long enough for a trailing fork to catch up, leading to rear-ending. Although many replisome components associate only transiently, the DnaB helicase stably associates with replisomes for tens of minutes during replication [63]. This contradicts collision models that rely on fork remodeling or enzymatic access to Y-shaped DNA like *recG/ruvAB*-mediated fork regression [47, 64] or *ruvC/rusA* fork cleavage [54], as the former would compete with DnaB occupancy and the latter would liberate DnaB from its bound ssDNA. Consistent with this idea, ChIP-qPCR of *B. subtilis* replicative helicase DnaC shows that replisomes stall and accumulate at sites of replicative stress [65] and linear DNA formed after Tus-induced fork stalling comprises newly replicated DNA rather than the parental template [49]. Therefore, replisome barriers, such as TRCs, result in replisome stalling and fork rear-ending. Repair pathways that depend on fork remodeling [4] are likely relegated to situations with more drastic replisome defects. A more detailed discussion of the context-dependence of TRCs and reported TRC outcomes can be found in Cooke *et al.* (2025) [66].

These results agree with previous observations that replisome barriers are a limiting factor in the viability of rapidly-replicating bacterial cells. Extensive work has shown that replication in the absence of the accessory replicative helicase, Rep, is slow and frequently disrupted by RNA polymerase barriers [11]. It appears that these barriers primarily induce stalling of the replisome and are bypassed through the activity of Rep [11, 67, 68]. When bypass of these barriers is impaired by *rep* deletion, cells become dependent on dsDNA resection by the RecBCD complex—as replicative DNA ends are generated as a result of increased replisome stalling [69]. Additional work has shown that replisome stalling appears to result in DNA damage that is greatly exacerbated by increases in the replication initiation rate. Disruptions in replisome elongation rate by oxidative lesions appear to be the driving force behind the nutrient sensitivity of hyper-initiating *Δhda* mutants [70] and hyper-initiating *ΔseqA* mutants acquire large amounts of spontaneous DNA damage [71]. Though it is worth noting that the DNA damage and possible fork rear-ending observed in *ΔseqA* cells was attributed to a post-replication spacer function of SeqA [72]. Nonetheless, DNA damage observed in *ΔseqA* cells has also been observed undergoing extensive DNA degradation [71], consistent with the replication reset model. This agreement with previous literature fortifies our findings as the methods used in these previous findings are not reliant on antibiotic treatment, and thus, are not subject to the off-target activities of antibiotics, such as alterations in nucleoid structure brought about by inhibiting transcription and translation [73], the effects on rRNA processing caused doxycycline [74], and the oxidative stress caused by aminoglycosides [75], such as kanamycin.

We find that the DNA damage response and homologous recombination are not activated by fork rear-ending. Conventional models propose that DNA ends arising from transcription–replication collisions activate homologous recombination, forming a chromosomal D-loop to recruit the primosome and restart replication. In contrast, our data indicate otherwise, consistent with prior observations that *E. coli* does not initiate the SOS response under sub-inhibitory translational stress [76] and that *dnaAcos* cells rely on *recB* but not *recA* for survival [77]. One possible explanation for absence of recombination is that it blocks inheritance of truncated linear chromosomes in daughter cells, which are nonviable, as shown for *E. coli recBC* mutants [78]. Another possibility is linear fragments are counter-selected because they massively amplify origin-proximal gene dosage, especially rRNA operons, titrating RNA polymerase away from essential genes. With fork rear-ending and resection blocked, replication continues without division, pushing rRNA operon copies to > 100 and creating an intolerable dosage burden [79, 80]. The specific mechanism blocking homologous recombination remains to be elucidated. *Escherichia coli* has multiple regulatory layers to control homologous recombination initiation, including *chi* recognition by RecBCD, RecA inhibitors such as RecX [81], and anti-RecA-filament helicases like UvrD [82]. Given that RecBCD slows upon encountering *chi* [83], potentially hindering rapid DNA resection, and a lack of damage-induced SOS activation, we hypothesize that recombination is inhibited at the step of *chi* recognition. This lack of SOS activation and homologous recombination likely explains the lack of mutagenesis attributed to TRCs [84, 85].

In bacteria that can initiate multiple replication forks, TRCs leading to DNA ends are considerably more frequent due to the increased number of active replisomes, as we showed for *E. coli* growing in nutrient-rich conditions. In these cells, the risk of illegitimate recombination also rises, as more replication forks increase the likelihood of introducing DNA ends into regions of homology. Timing is also critical; on average, break repair requires about 15 min and risks overlapping with an approaching replisome [88]. In this scenario, a rapid, large-scale degradation-based “reset” can be advantageous, quickly removing collisions and avoiding both hyper-recombination events and the additional collisions that could arise from delayed break repair. By contrast, bacteria that typically maintain only a single round of replication forks, in nutrient-poor environments, may use replication fork restart/recombination more readily. In these organisms, extended transcription-stalled replisomes may regress or collapse, making homology-directed repair a more efficient strategy. Thus, TRC resolution strategies differ between multifork and single-fork replication lifestyles, reflecting each organism’s balance between minimizing recombination-based errors and the energetic cost of DNA degradation. Similar risks of illegitimate recombination have been observed in eukaryotes, where homologous recombination substrates are formed at sites of replisome fusion, resulting in mutation [87] when not degraded by processive nucleases [86]. This suggests that antihomologous recombination mechanisms may be conserved across organisms to avoid the mutagenic consequences of replicative failures.

## Supplementary Material

gkaf1227_Supplemental_Files

## Data Availability

Further information and requests for data, resources, and reagents should be directed to and will be fulfilled by the lead contact, Dr Christophe Herman (herman@bcm.edu). Bacterial strains and plasmids generated in this study are available upon request. Raw sequencing data has been deposited to the Sequence Read Archive and is publicly available as of the date of publication. SRA accession numbers are listed in the key resource table and Supplementary Table S7. Microscopy images, flow cytometry FCS files, CFU counts, intermediate NGS data files, and any other data relevant to this publication are available upon request. Any additional information required to reanalyze the data reported in this paper is available from the lead contact upon request. All original code has been deposited to our lab Github page and is publicly available as of the date of publication. DOIs are listed in the key resources table.
