# Supplementary material for: Transcription–replication collisions trigger high-fidelity replication reset
Source: Nucleic Acids Res. 2025 Nov 26;53(22):gkaf1227. doi: 10.1093/nar/gkaf1227 (PMC12651559; doi:10.1093/nar/gkaf1227)
Supplement: gkaf1227_Supplemental_Files [file gkaf1227_supplemental_files.zip › Revisions_Supplement.docx]

**Supplementary Material**

**Supplementary Figures**


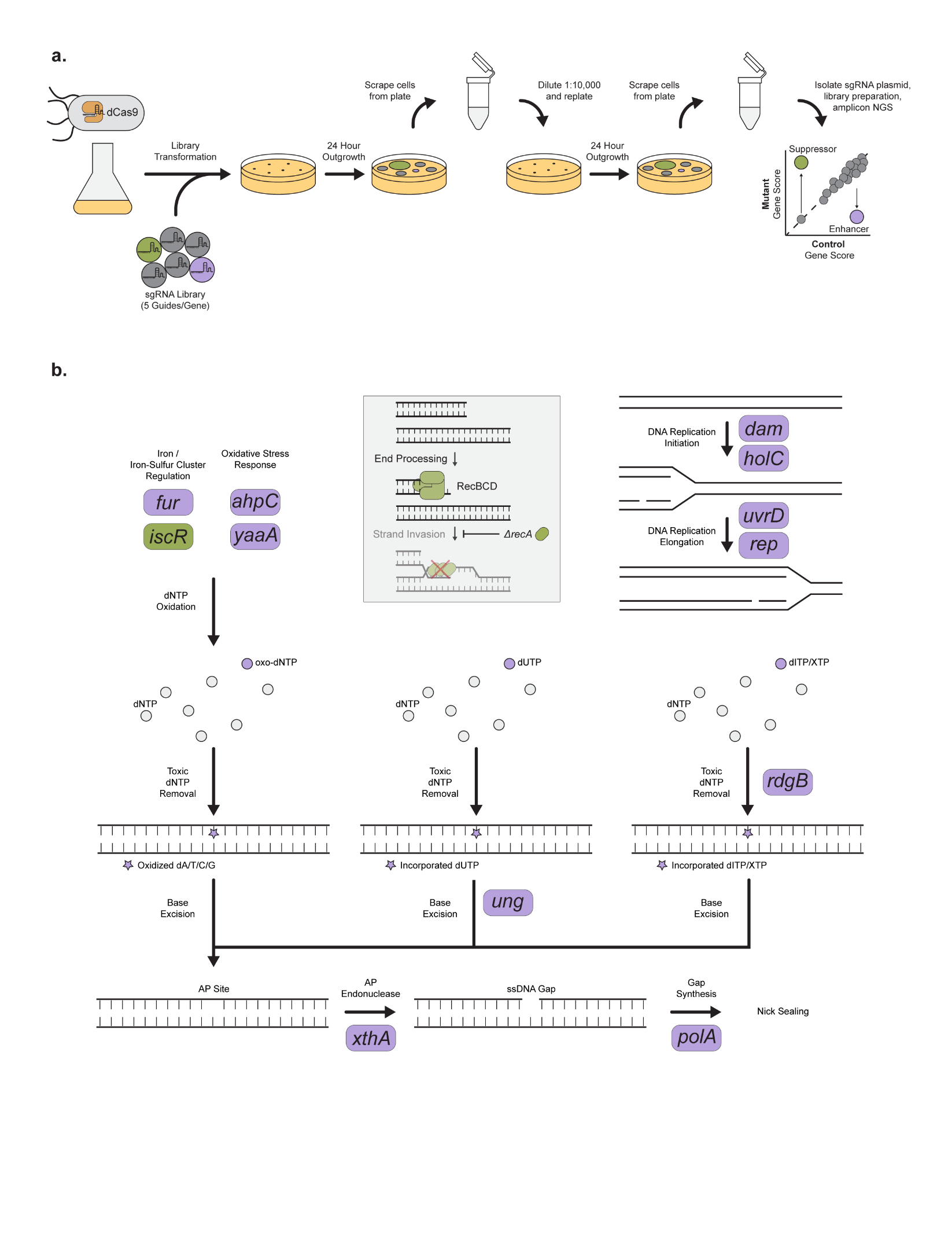


**Supplementary Figure 1 – Visual representation of CRISPRi-screening approach.**

a. Schematic of CRISPRi-screening. Cells that inducibly express dCas9 are transformed with a library of sgRNA plasmids that target each gene in the genome. Transformants are plated onto growth media containing the dCas9 inducer. A 24-hour outgrowth allows cells to increase or decrease in relative abundance as a function of sgRNA-induced growth rate increases or decreases. Cells are scraped from the first plate, diluted, and re-plated onto plates containing the dCas9 inducer for a second round of outgrowth. The final cells are scraped from the second outgrowth plate and sgRNA plasmids are extracted from the cell pool. This plasmid is prepared for next-generation sequencing and the relative abundances of sgRNA plasmids are compared to a control condition to identify sgRNAs that conferred a growth advantage or disadvantage to the genotype of interest. b. Visual representation of the major interactions between the *ΔrecA* genetic background and sgRNA-mediated knockdowns in Figure 1d. Results recapitulate known synthetic lethality and growth defects between *ΔrecA* and disruptions to DNA replication initiation/elongation, as well as repair disruption of dsDNA base damage/misincorporations. Gene names from Figure 1d are labelled as green for knockdowns that result in a growth advantage and purple for knockdowns that result in a growth disadvantage.


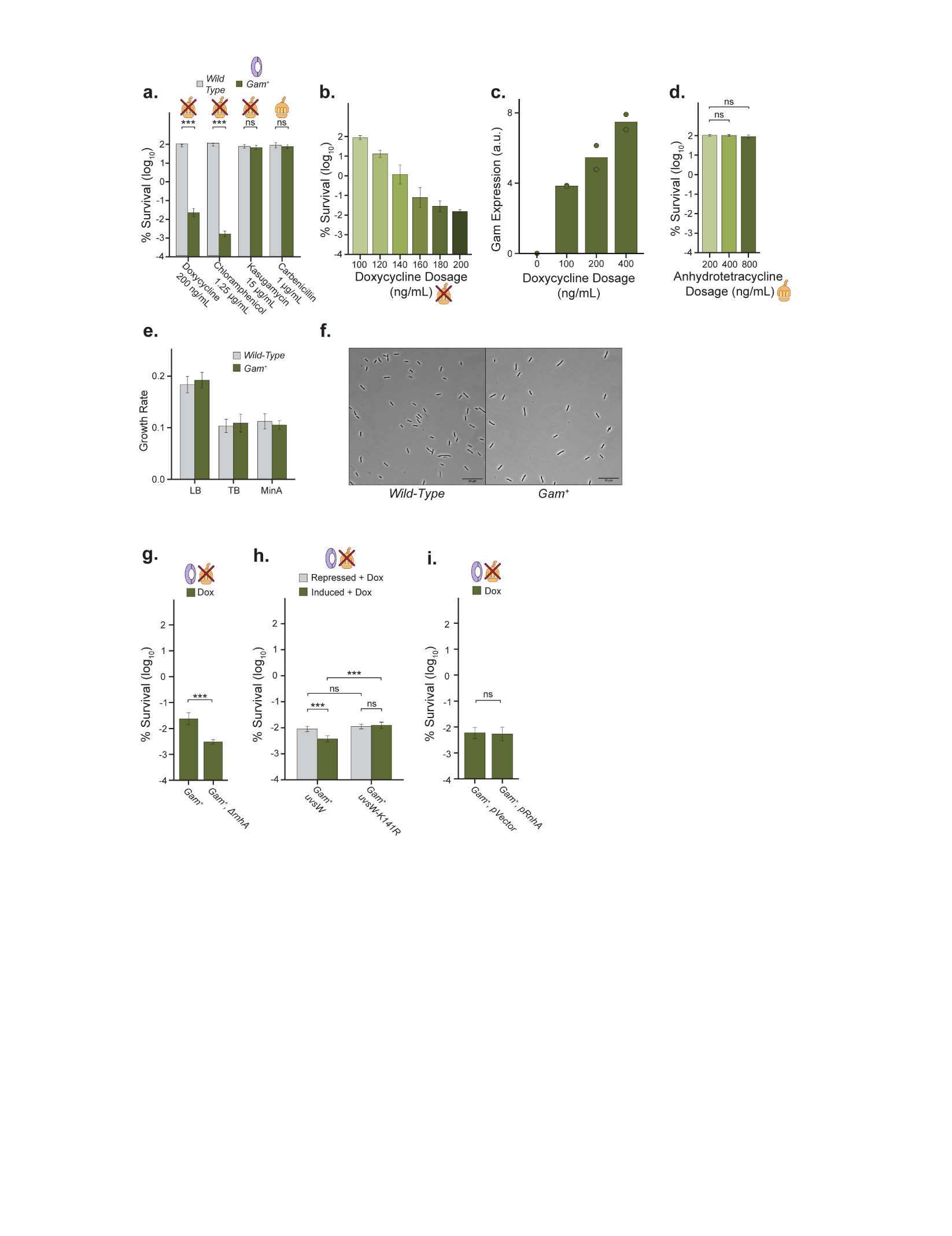


**Supplementary Figure 2 – Gam-dependent DNA end phenotypes are not explained by inherent toxicity or R-loop activity.**

a. Plating C.F.U. of wild-type or *gam*-expressing cells onto the indicated antibiotic, with *gam*-inducer, relative to titer. (N=9; 95% CI). b. Dose-response curve of *gam*-induced doxycycline lethality (N=9; 95% CI). c. Quantification of relevant anti-Gam Western blots (Fig. S3), relative to anti-GroEL control, as a function of doxycycline dose (N=2). d. Increasing levels of non-ribosome binding *gam*-inducer, anhydrotetracycline, does not result in *gam*-dependent lethality. (N=9; 95% CI). e. Quantification of exponential phase growth rate of wild-type and *gam^+^* cells in the three media types used in this study show no *gam*-dependent growth defect in the absence of translation stress (N=12; 95% CI). f. Representative phase contrast micrographs of exponential phase wild-type and *gam^+^* cells (Scale bars = 20 µm). g. Percent viable cells of the indicated genotypes plated on 200 ng/mL doxycycline (N=9; 95% C.I.). h. Percent viable cells of the indicated genotypes plated on 200 ng/mL doxycycline ± 100 µM IPTG (N=9; 95% C.I.). i. Percent viable cells of the indicated genotypes plated on 200 ng/mL doxycycline, while maintaining plasmid (N=9; 95% C.I.).


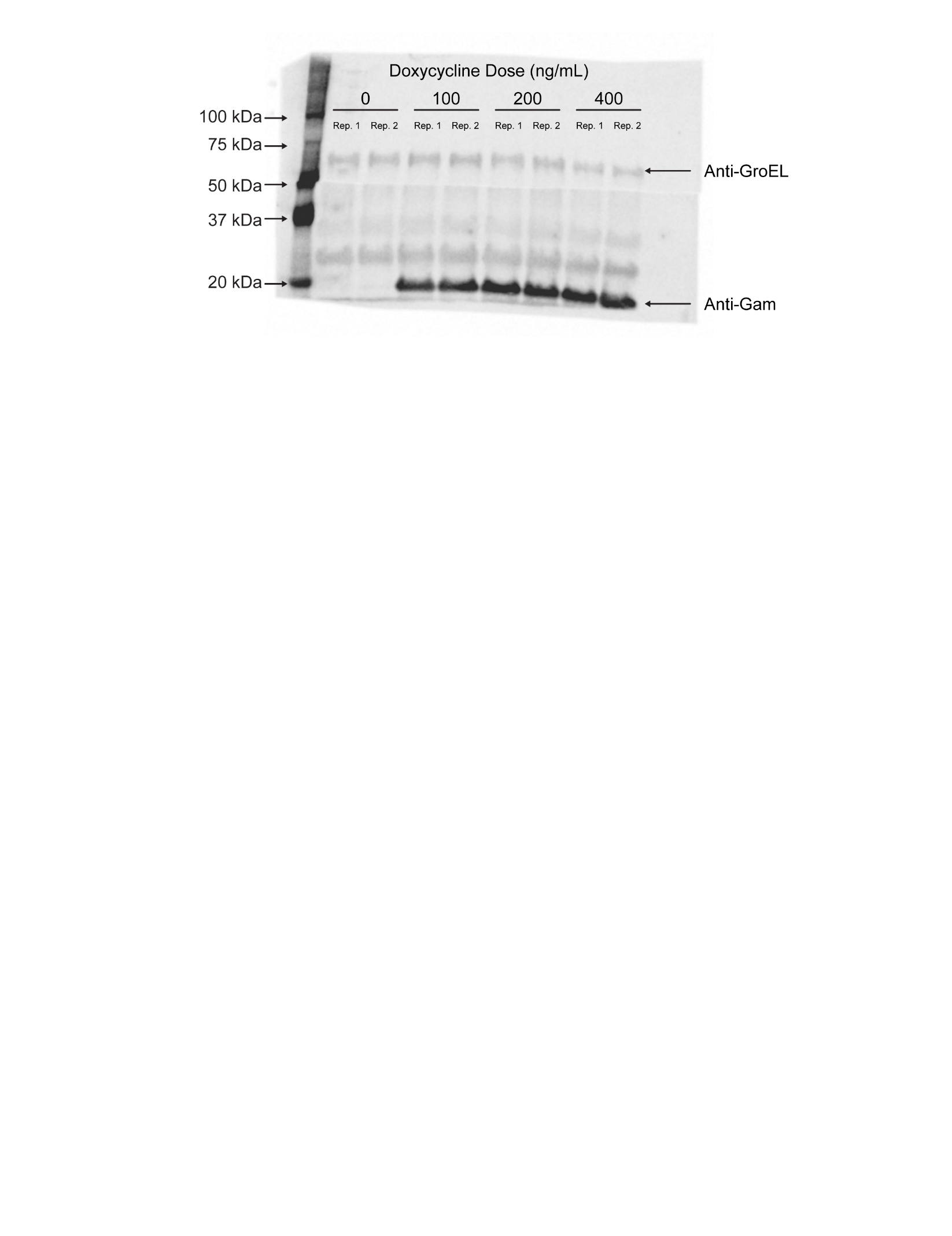


**Supplementary Figure 3 – Raw Gam western blot corresponding to Supplementary Figure 2C.**

Gam levels were quantified as a function of doxycycline dosage and normalized to GroEL levels.


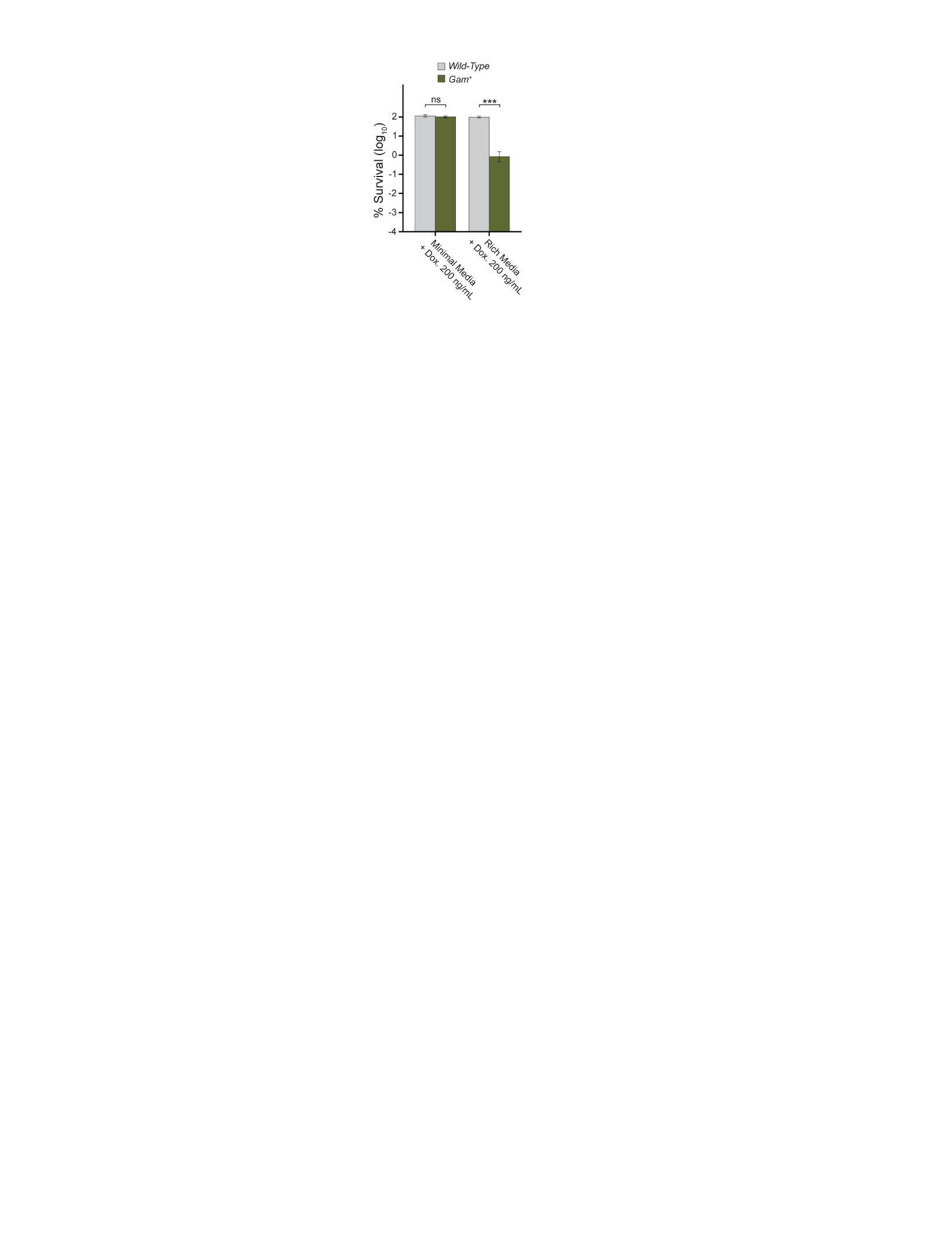


**Supplementary Figure 4 – *gam*-induced translation inhibitor sensitivity is a function of media richness.**

*gam*-induced killing in translation-stressed cells as a function of media richness. Cell survival with 200 ng/mL doxycycline in minimal or LB media for wild-type and *gam^+^* cells (N=9, 95% CI).


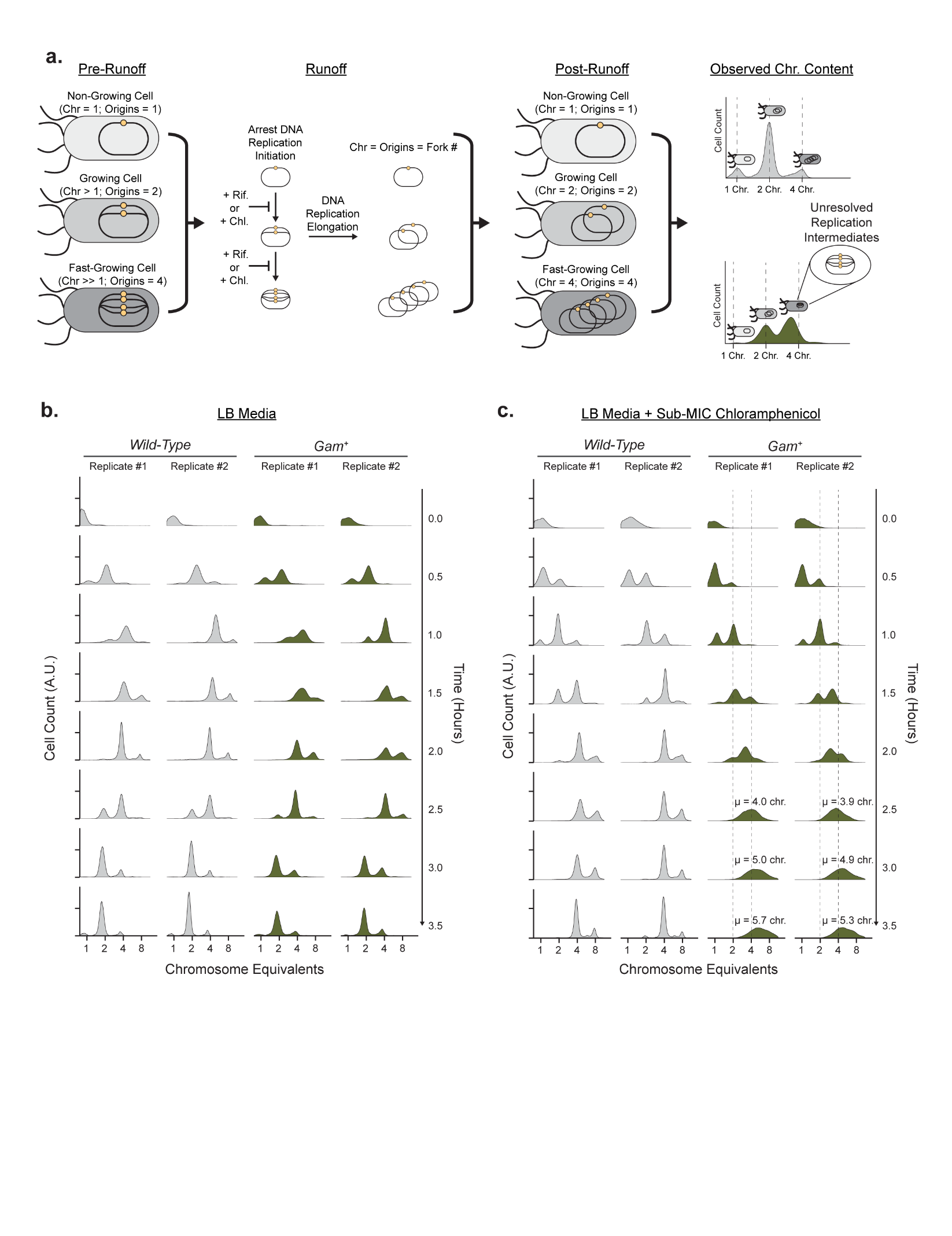


**Supplementary Figure 5 – Translation disruption causes accumulation of DNA replication intermediates.**

a. Schematic explanation of replication run-out assay and run-out assay data readout. Prior to replication run-out, the cell population is a mix of cells in non-replicating (light grey), replicating (grey), and fast replicating (dark grey) states. These different growth stages contain a mixture of chromosome abundances that only roughly correlate to their replication state. During the run-out, transcription (high-dose rifampicin) or translation (high-dose chloramphenicol) are inhibited to arrest the initiation of DNA replication. Additionally, cephalexin is added to inhibit cell division, which would confound DNA content per cell measurements. At the end of the run-out, cells will contain a distinct, integer number of chromosomes that reveal the cell replication state at the time of initiating the run-out. Histograms of chromosome abundance (DAPI signal) reveal the replicative behavior of the cell population. Chromosome counts corresponding to 2^n^ (with ‘n’ corresponding to active replication cycles) reveal completed replication cycles during the run-out. Post-run-out chromosome counts that do not conform to 2^n^ indicate incomplete chromosome replication cycles or DNA damage intermediates. b. Raw time-course replication run-out experiments demonstrate that wild-type and *gam^+^* cells do not accumulate incomplete replication intermediates during unstressed growth. c. Raw time course replication run-out experiments demonstrate that *gam^+^* cells accumulate incomplete replication intermediates when stressed by sub-inhibitory doses of translation elongation inhibitor.

**
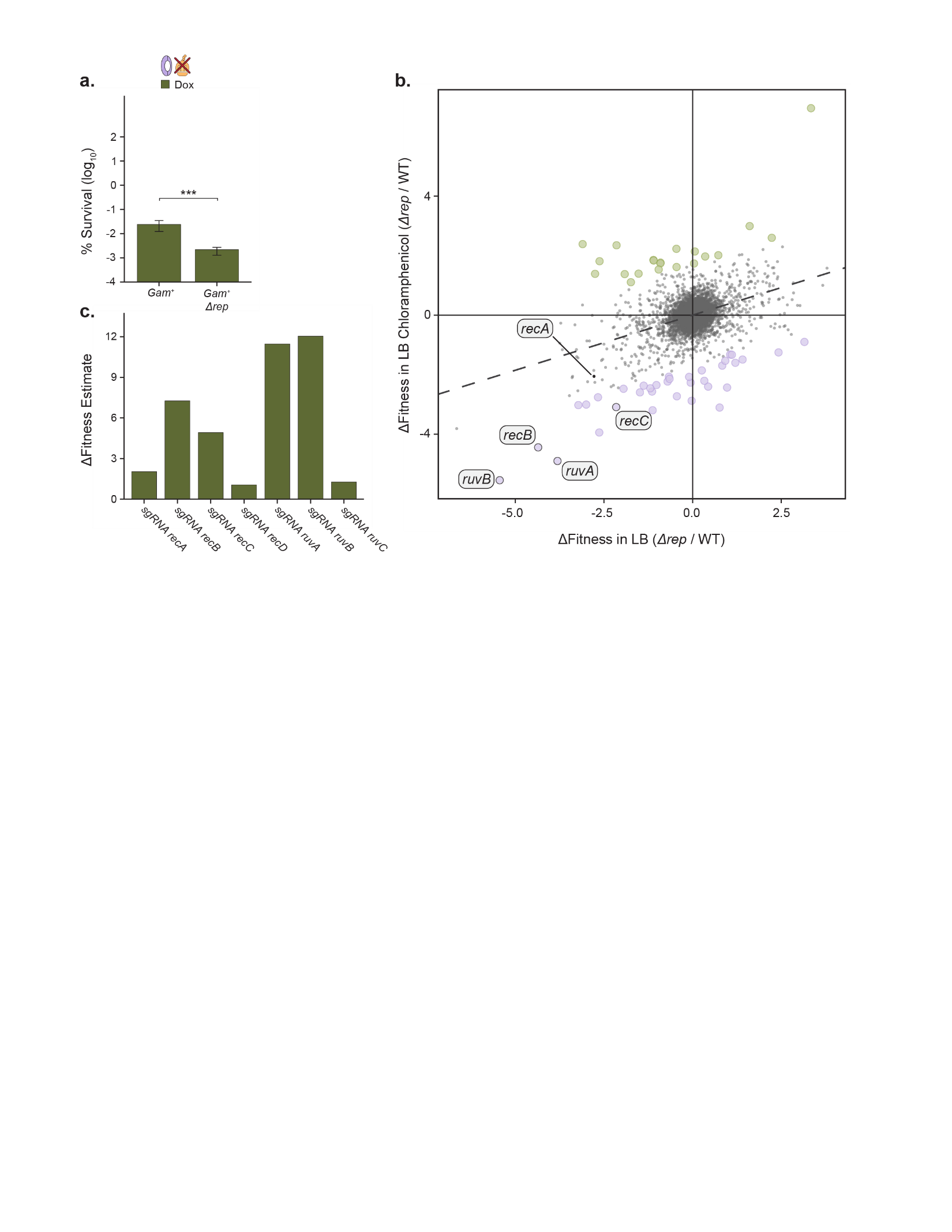
**

**Supplementary Figure 6 – *Δrep*-induced TRCs cause a dependency on the RuvABC and RecBCD complexes.**

a. Plating C.F.U. (derived from Figure 1k) of *gam*-expressing cells on 200 ng/mL doxycycline, relative to titer. (N=9; 95% CI). b. CRISPRi phenotypic profiling of Wild-type cells’ genetic dependencies on LB 1.25 μg/mL chloramphenicol relative to LB (x-axis), compared to *Δrep* genetic dependencies on LB 1.25 μg/mL chloramphenicol relative to LB (y-axis). (N=4). c. Log_2_FC fitness estimates for the indicated gene knockdowns derived from panel b.


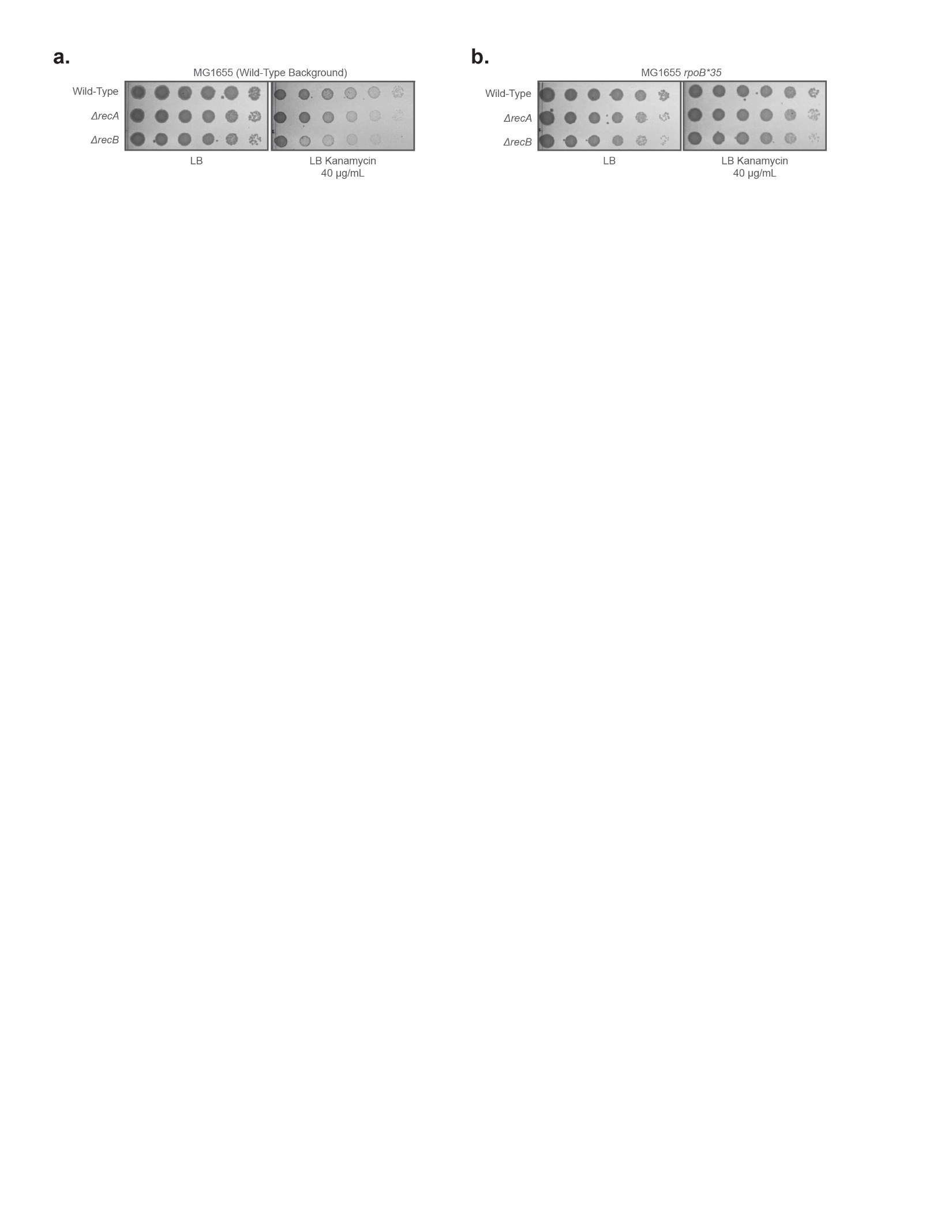


**Supplementary Figure 7 – Loss of RecBCD nuclease activity leads to a kanamycin sensitivity.** a.) The indicated genotypes containing a kanamycin resistance cassette were plated on LB control or LB 40 μg/mL kanamycin A in wild-type (a.) or *rpoB*35* (b.) backgrounds.

**Supplementary Tables**

**Supplementary Table 5 – Strains used in this study.**

| Strain | Genotype | Figure | Origin |
| --- | --- | --- | --- |
| Figure 1 | | | |
| 8201 | MG1655 attB186::PtetA-dCas9::FRT | 1d,e | This study |
| 8202 | MG1655 attB186::PtetA-dCas9::FRT | 1d,e | This study |
| 8670 | MG1655 attB186::PtetA-dCas9::FRT, ΔrecA::FRT | 1d,f,g | This study |
| 8671 | MG1655 attB186::PtetA-dCas9::FRT, ΔrecA::FRT | 1d,f,g | This study |
| 9336 | MG1655 attB186::PtetA-dCas9::FRT, attTn7::PN25tetO-gam::FRT | 1e,f,g | This study |
| 9337 | MG1655 attB186::PtetA-dCas9::FRT, attTn7::PN25tetO-gam::FRT | 1e,f,g | This study |
| 13694 | MG1655 yfeP::PN25-tetR::FRT, attTn7::PN25tetO-gam::FRT, ΔrsmD::FRT | 1h | This study |
| 14809 | MG1655 yfeP::PN25-tetR::FRT, attTn7::PN25tetO-gam::FRT, ΔtrmH::FRT | 1h | This study |
| 14813 | MG1655 yfeP::PN25-tetR::FRT, attTn7::PN25tetO-gam::FRT, Δtgt::FRT | 1h | This study |
| 13692 | MG1655 yfeP::PN25-tetR::FRT, attTn7::PN25tetO-gam::FRT, ΔtruB::FRT | 1h | This study |
| 14810 | MG1655 yfeP::PN25-tetR::FRT, attTn7::PN25tetO-gam::FRT, ΔproQ::FRT | 1h | This study |
| 9560 | MG1655 yfeP::PN25-tetR::FRT, attTn7::PN25tetO-gam::FRT, ΔssrA::FRT | 1h | This study |
| 9620 | MG1655 yfeP::PN25-tetR::FRT, attTn7::PN25tetO-gam::FRT, ΔsmpB::FRT | 1h | This study |
| 30 | MG1655 | 1j | Lab Stock |
| 4619 | MG1655 ΔrecA::Kan:FRT | 1j | Ref^1^ |
| 4252 | MG1655 yfeP::PN25-tetR::FRT, attTn7::PN25tetO-gam::FRT | 1j,k | This study |
| 9515 | MG1655 yfeP::PN25-tetR::FRT, attTn7::PN25tetO-gam::FRT, rpsL^1204^ | 1j | This study |
| 10553 | MG1655 yfeP::PN25-tetR::FRT, attTn7::PN25tetO-gam::FRT, Δrep::FRT | 1k | This study |
| 5069 | MG1655 yfeP::PN25-tetR::FRT, attTn7::PN25tetO-gam::FRT, ΔdksA::Kan:FRT | 1k | This study |
| 10105 | MG1655 yfeP::PN25-tetR::FRT, attTn7::PN25tetO-gam::FRT, rpoB*35::FRT | 1k | This study |
| Figure 2 | | | |
| 30 | MG1655 | 2a,b,c | Lab Stock |
| 4252 | MG1655 yfeP::PN25-tetR::FRT, attTn7::PN25tetO-gam::FRT | 2a,b,c,e | This study |
| 9558 | MG1655 yfeP::PN25-tetR::FRT, attTn7::PN25tetO-gam::FRT, ΔdiaA::FRT | 2e | This study |
| 10131 | MG1655 yfeP::PN25-tetR::FRT, attTn7::PN25tetO-gam::FRT, ΔdiaA::FRT, Δhda::Kan:FRT | 2e | This study |
| 13054 | MG1655 yfeP::PN25-tetR::FRT, attTn7::PN25tetO-gam::FRT, pDS596 | 2f | This study |
| 13056 | MG1655 yfeP::PN25-tetR::FRT, attTn7::PN25tetO-gam::FRT, pLS120 | 2f | This study |
| Figure 3 | | | |
| 4252 | MG1655 yfeP::PN25-tetR::FRT, attTn7::PN25tetO-gam::FRT | 3a,b | This study |
| 12432 | MG1655 I-4::Kan:FRT | 3c,e | This study |
| 12442 | MG1655 I-4::Kan:FRT, rpoB*35 | 3c | This study |
| 12434 | MG1655 ΔrecA::Kan:FRT | 3c | This study |
| 12444 | MG1655 ΔrecA::Kan:FRT, rpoB*35 | 3c | This study |
| 11328 | MG1655 ΔrecB::Kan:FRT | 3c,f | This study |
| 11338 | MG1655 ΔrecB::Kan:FRT, rpoB*35 | 3c | This study |
| 12438 | MG1655 ΔrecD::Kan:FRT | 3c | This study |
| 12448 | MG1655 ΔrecD::Kan:FRT, rpoB*35 | 3c | This study |
| 5344 | MG1655 ΔrecJ::Kan:FRT | 3d | Ref^1^ |
| 14807 | MG1655 ΔrecJ::Kan:FRT, rpoB*35 | 3d | This study |
| 14791 | MG1655 ΔrecJ::FRT, ΔrecA::Kan:FRT | 3d | This study |
| 14851 | MG1655 ΔrecJ::FRT, ΔrecA::Kan:FRT, rpoB*35 | 3d | This study |
| 14792 | MG1655 ΔrecJ::FRT, ΔrecB::Kan:FRT | 3d,g | This study |
| 14852 | MG1655 ΔrecJ::FRT, ΔrecB::Kan:FRT, rpoB*35 | 3d | This study |
| 14794 | MG1655 ΔrecJ::FRT, ΔrecD::Kan:FRT | 3d | This study |
| 14854 | MG1655 ΔrecJ::FRT, ΔrecD::Kan:FRT, rpoB*35 | 3d | This study |
| Figure 4 | | | |
| 12470 | MG1655 yfeP::PN25-tetR::FRT, attTn7::PN25tetO-Gam::FRT, invD, ΔybfF::Kan:FRT | 4b,d | This study |
| 9617 | MG1655 yfeP::PN25-tetR::FRT, attTn7::PN25tetO-MuGam::FRT, ΔseqA::FRT, invD | 4b,c,d | This study |
| 12493 | MG1655 yfeP::PN25-tetR::FRT, attTn7::PN25tetO-MuGam::FRT, ΔseqA::FRT, invD, rpoB*35::Kan:FRT | 4b,d | This study |
| 12496 | MG1655 yfeP::PN25-tetR::FRT, attTn7::PN25tetO-MuGam::FRT, ΔseqA::FRT, invD, ΔdiaA::Kan:FRT | 4b,d | This study |
| 4252 | MG1655 yfeP::PN25-tetR::FRT, attTn7::PN25tetO-gam::FRT | 4e | This study |
| 10681 | MG1655 yfeP::PN25-tetR::FRT, attTn7::PN25tetO-gam::FRT, ΔruvA::FRT | 4e | This study |
| 10683 | MG1655 yfeP::PN25-tetR::FRT, attTn7::PN25tetO-gam::FRT, ΔruvB::FRT | 4e | This study |
| 7653 | MG1655 yfeP::PN25-tetR::FRT, attTn7::PN25tetO-gam::FRT, ΔruvC::Cam | 4e | This study |
| 11957 | MG1655 yfeP::PN25-tetR::FRT, attTn7::PN25tetO-gam::FRT, ΔrecG::FRT | 4e | This study |
| 12432 | MG1655 I-4::Kan:FRT | 4f | This study |
| 12442 | MG1655 I-4::Kan:FRT, rpoB*35 | 4f | This study |
| 11322 | MG1655 ΔruvA::Kan:FRT | 4f | This study |
| 11332 | MG1655 ΔruvA::Kan:FRT, rpoB*35 | 4f | This study |
| 11324 | MG1655 ΔruvB::Kan:FRT | 4f | This study |
| 11334 | MG1655 ΔruvB::Kan:FRT, rpoB*35 | 4f | This study |
| 11326 | MG1655 ΔruvC::Kan:FRT | 4f | This study |
| 11336 | MG1655 ΔruvC::Kan:FRT, rpoB*35 | 4f | This study |
| 11320 | MG1655 ΔrecG::Kan:FRT | 4f | This study |
| 11330 | MG1655 ΔrecG::Kan:FRT, rpoB*35 | 4f | This study |
| Figure 5 | | | |
| 5958 | MG1655 pUA66-PsulA-GFPmut2 | 5a,b,h | Ref^1^ |
| 30 | MG1655 | 5c,g,i | Lab Stock |
| 6677 | MG1655 rpoB*35 | 5c,g | Ref^1^ |
| 15663 | FC40 attλ::ampRC | 5d | Ref^2^ |
| 15695 | FC40 attλ::ampRC, rpoB*35 I-84::FRT | 5d | This study |
| 7245 | MG1655 attλ::cI(ind-)-tetA | 5e | This study |
| 15691 | MG1655 attλ::cI(ind-)-tetA, rpoB*35 I-84::FRT | 5e | This study |
| 10719 | MG1655 Δrep::FRT | 5g | This study |
| 15726 | MG1655 Δrep::FRT, rpoB*35 | 5g | This study |
| 11328 | MG1655 ΔrecB::Kan:FRT | 5i | This study |
| 14792 | MG1655 ΔrecB::Kan:FRT, ΔrecJ::FRT | 5i | This study |
| 15148 | MG1655 recB^D1080A^ argA::TN10, pUA66-PsulA-GFPmut2 | 5h | This study |
| 6224 | MG1655 recB^D1080A^ argA::TN10 | 5h | Ref^1^ |
| Figure 6 | | | |
| None |  |  |  |
| Supplementary Figure 1 | | | |
| None |  |  |  |
| Supplementary Figure 2 | | | |
| 4252 | MG1655 yfeP::PN25-tetR::FRT, attTn7::PN25tetO-gam::FRT | S2a-g | This study |
| 30 | MG1655 | S2e,f | Lab Stock |
| 10256 | MG1655 yfeP::PN25-tetR::FRT, attTn7::PN25tetO-gam::FRT, ΔrnhA::FRT | S2g | This study |
| 10224 | MG1655 yfeP::PN25-tetR::FRT, attTn7::PN25tetO-gam::FRT, attλ::Ptac-uvsW::Amp | S2h | This study |
| 10225 | MG1655 yfeP::PN25-tetR::FRT, attTn7::PN25tetO-gam::FRT, attλ::Ptac-uvsW^K141R^::Amp | S2h | This study |
| 13630 | MG1655 yfeP::PN25-tetR::FRT, attTn7::PN25tetO-gam::FRT, pSK762 | S2i | This study |
| 13632 | MG1655 yfeP::PN25-tetR::FRT, attTn7::PN25tetO-gam::FRT, pSK760 | S2i | This study |
| Supplementary Figure 3 | | | |
| 4252 | MG1655 yfeP::PN25-tetR::FRT, attTn7::PN25tetO-gam::FRT | S3 | This study |
| Supplementary Figure 4 | | | |
| 30 | MG1655 | S4 | Lab Stock |
| 4252 | MG1655 yfeP::PN25-tetR::FRT, attTn7::PN25tetO-gam::FRT | S4 | This study |
| Supplementary Figure 5 | | | |
| 30 | MG1655 | S5b,c | Lab Stock |
| 4252 | MG1655 yfeP::PN25-tetR::FRT, attTn7::PN25tetO-gam::FRT | S5b,c | This study |
| Supplementary Figure 6 | | | |
| 4252 | MG1655 yfeP::PN25-tetR::FRT, attTn7::PN25tetO-gam::FRT | S6a | This study |
| 10553 | MG1655 yfeP::PN25-tetR::FRT, attTn7::PN25tetO-gam::FRT, Δrep::FRT | S6a | This study |
| 8201 | MG1655 attB186::PtetA-dCas9::FRT | S6b,c | This study |
| 8202 | MG1655 attB186::PtetA-dCas9::FRT | S6b,c | This study |
| 11578 | MG1655 attB186::PtetA-dCas9::FRT, Δrep::FRT | S6b,c | This study |
| 11580 | MG1655 attB186::PtetA-dCas9::FRT, Δrep::FRT | S6b,c | This study |
| Supplementary Figure 7 | | | |
| 12432 | MG1655 I-4::Kan:FRT | S7a | This study |
| 12434 | MG1655 ΔrecA::Kan:FRT | S7a | This study |
| 11328 | MG1655 ΔrecB::Kan:FRT | S7a | This study |
| 12442 | MG1655 I-4::Kan:FRT, rpoB*35 | S7b | This study |
| 12444 | MG1655 ΔrecA::Kan:FRT, rpoB*35 | S7b | This study |
| 11338 | MG1655 ΔrecB::Kan:FRT, rpoB*35 | S7b | This study |

**Supplementary Table 6 – Strain Building Table**

|  | | | | |
| --- | --- | --- | --- | --- |
| Strain | Parent | Background | Manipulation/Source | Genotype |
| - | - | BW25113 | Keio Collection | ΔdksA::Kan:FRT |
| - | - | BW25113 | Keio Collection | ΔdiaA::Kan:FRT |
| - | - | MG1655 | Nehring et al. 2015 | I-84::Kan:FRT |
| CH30 | N/A | MG1655 | Lab Stock |  |
| CH121 | N/A | HME45 | Lab Stock |  |
| CH543 | N/A | MG1655 | Lab Stock | lacZ^U118^ |
| CH560 | N/A | BW25113 | Keio Collection | ΔgalK::Kan:FRT |
| CH580 | CH543 | MG1655 | P1 Transduction (Keio dksA) | lacZ^U118^, ΔdksA::Kan:FRT |
| CH603 | CH30 | MG1655 | P1 Transduction (CH560) | ΔgalK::Kan:FRT |
| CH3055 | N/A | W3110 | Barral Lab | rpsL^1204^::TN10 |
| CH3782 | N/A | MG1655 | Susan Rosenberg Lab | araBad567, attλ::pBAD Zfd2509.2::PN25-tetR::FRT, attTn7:: PN25-gam-GFP::Cam:FRT |
| CH3837 | N/A | BW25113 | Keio Collection | ΔrecG::Kan:FRT |
| CH3929 | CH3782 | MG1655 | P1 Transduction (Keio recB) | araBad567, attλ::pBAD Zfd2509.2::PN25-tetR::FRT, attTn7:: PN25-gam-GFP::Cam:FRT, recB::Kan:FRT |
| CH4048 | N/A | JA200 | Saka et al. 2005 | pNT3-recA |
| CH4088 | CH4048 | BW25113 | Conjugation (CH4048) | ΔrecA::Kan:FRT, pNT3-recA |
| CH4199 | N/A | MG1655 | Susan Rosenberg Lab | attλ::PN25-tetR::FRT, attTn7::PN25tetO-gam::Cam:FRT |
| CH4200 | N/A | FC40 | Susan Rosenberg Lab | yfeP::PN25-tetR::Kan:FRT |
| CH4230 | CH30 | MG1655 | P1 Transduction (CH4200) | yfeP::PN25-tetR::Kan:FRT |
| CH4242 | CH4230 | MG1655 | P1 Transduction (CH4199) | yfeP::PN25-tetR::Kan:FRT, attTn7::PN25tetO-gam::Cam:FRT |
| CH4252 | CH4242 | MG1655 | Marker Flip (pCP20) | yfeP::PN25-tetR::FRT, attTn7::PN25tetO-gam::FRT |
| CH4581 | N/A | JA200 | Saka et al. 2005 | pNT3-recB |
| CH4619 | CH30 | MG1655 | P1 Transduction (CH4088) | ΔrecA::Kan:FRT |
| CH4760 | N/A | BW25113 | Keio Collection | ΔruvC::Kan:FRT |
| CH4788 | CH30 | MG1655 | P1 Transduction (CH3837) | ΔrecG::Kan:FRT |
| CH4844 | N/A | BW25113 | Keio Collection | ΔrnhA::Kan:FRT |
| CH5069 | CH4252 | MG1655 | P1 Transduction (CH580) | yfeP::PN25-tetR::FRT, attTn7::PN25tetO-gam::FRT, ΔdksA::Kan:FRT |
| CH5097 | CH3929 | MG1655 | Conjugation (CH4581) | araBad567, attλ::pBAD Zfd2509.2::PN25-tetR::FRT, attTn7:: PN25-gam-GFP::Cam:FRT, recB::Kan:FRT, pNT3-recB |
| CH5333 | N/A | BW25113 | Keio Collection |  |
| CH5344 | CH30 | MG1655 | P1 Transduction (CH5333) | ΔrecJ::Kan:FRT |
| CH5689 | N/A | BW25113 | Keio Collection | ΔruvB::Kan:FRT |
| CH5958 | CH30 | MG1655 | Transformation | pUA66-PsulA-GFPmut2 |
| CH6083 | CH30 | MG1655 | P1 Transduction (Keio diaA) | ΔdiaA::Kan:FRT |
| CH6178 | N/A | 11B 360 | Ana Simatovic Lab | recB^D1080A^ argA::TN10 |
| CH6211 | CH6178 | 11B 360 | Conjugation (CH4581) | recB^D1080A^ argA::TN10, pNT3-recB |
| CH6224 | CH30 | MG1655 | P1 Transduction (CH6211) | recB^D1080A^ argA::TN10 |
| CH6654 | N/A | MG1655 | Bob Lloyd Lab (N5530) | rpoB*35, argE86::TN10 |
| CH6658 | N/A | MG1655 | Bob Lloyd Lab (N4849) | rpoB*35 |
| CH6660 | CH30 | MG1655 | P1 Transduction (CH6654) | argE86::TN10 |
| CH6677 | CH6660 | MG1655 | P1 Transduction (CH6658) | rpoB*35 |
| CH6720 | N/A | BW25113 | Keio Collection | ΔseqA::Kan:FRT |
| CH7014 | N/A | MG1655 | Jay Gowrishankar Lab | (argF-lac)U169, attλ::Ptac-uvsW::Amp |
| CH7015 | N/A | MG1655 | Jay Gowrishankar Lab | (argF-lac)U169, attλ::Ptac-uvsW^K141R^::Amp |
| CH7194 | N/A | MG1655 | David Bikard Lab (LC-E75) | attλ::mCherry, att186::dCas9 |
| CH7245 | CH603 | MG1655 | P1 Transduction | attλ::cI(ind-)-tetA |
| CH7607 | N/A | MG1655 | Susan Rosenberg Lab (SMR8899) | ruvC::Cam, pGBruvABC::Spc |
| CH7653 | CH4252 | MG1655 | P1 Transduction (CH7607) | yfeP::PN25-tetR::FRT, attTn7::PN25tetO-gam::FRT, ΔruvC::Cam |
| CH8112 | CH7194 | MG1655 | Recombineering (pKD46) | attλ::mCherry, att186::dCas9::Cam:FRT |
| CH8201 | CH30 | MG1655 | P1 Transduction (CH8112) | att186::dCas9::FRT |
| CH8202 | CH30 | MG1655 | P1 Transduction (CH8112) | att186::dCas9::FRT |
| CH8670 | CH8201 | MG1655 | P1 Transduction (CH4088) | attB186::PtetA-dCas9::FRT, ΔrecA::FRT |
| CH8671 | CH8202 | MG1655 | P1 Transduction (CH4088) | attB186::PtetA-dCas9::FRT, ΔrecA::FRT |
| CH9246 | N/A | MG1655 | Nehring et al. 2015 | I-4::Kan:FRT |
| CH9336 | CH8201 | MG1655 | P1 Transduction (CH4242) | attB186::PtetA-dCas9::FRT, attTn7::PN25tetO-gam::FRT |
| CH9337 | CH8202 | MG1655 | P1 Transduction (CH4242) | attB186::PtetA-dCas9::FRT, attTn7::PN25tetO-gam::FRT |
| CH9515 | CH4252 | MG1655 | P1 Transduction (CH3055) | yfeP::PN25-tetR::FRT, attTn7::PN25tetO-gam::FRT, rpsL^1204^::TN10 |
| CH9538 | N/A | BW25113 | Keio Collection | ΔsmpB::Kan:FRT |
| CH9539 | CH121 | HME45 | Recombineering | ΔssrA::Kan:FRT |
| CH9558 | CH4252 | MG1655 | P1 Transduction (CH6083) | yfeP::PN25-tetR::FRT, attTn7::PN25tetO-gam::FRT, ΔdiaA::FRT |
| CH9560 | CH4252 | MG1655 | P1 Transduction (CH9539) | yfeP::PN25-tetR::FRT, attTn7::PN25tetO-gam::FRT, ΔssrA::FRT |
| CH9617 | CH4252 | MG1655 | P1 Transduction (CH6720) | yfeP::PN25-tetR::FRT, attTn7::PN25tetO-MuGam::FRT, ΔseqA::FRT, invD |
| CH9620 | CH4252 | MG1655 | P1 Transduction (CH9538) | yfeP::PN25-tetR::FRT, attTn7::PN25tetO-gam::FRT, ΔsmpB::FRT |
| CH9964 | CH6677 | MG1655 | P1 Transduction (I-Deconvoluter 84) | rpoB*35, I-84::Kan:FRT |
| CH10018 | N/A | BW25113 | Keio Collection | Δhda::Kan:FRT |
| CH10105 | CH4252 | MG1655 | P1 Transduction (CH9964) | yfeP::PN25-tetR::FRT, attTn7::PN25tetO-gam::FRT, rpoB*35::FRT |
| CH10131 | CH9558 | MG1655 | P1 Transduction (CH10018) | yfeP::PN25-tetR::FRT, attTn7::PN25tetO-gam::FRT, ΔdiaA::FRT, Δhda::Kan:FRT |
| CH10224 | CH4252 | MG1655 | P1 Transduction (CH7014) | yfeP::PN25-tetR::FRT, attTn7::PN25tetO-gam::FRT, attλ::Ptac-uvsW::Amp |
| CH10225 | CH4252 | MG1655 | P1 Transduction (CH7015) | yfeP::PN25-tetR::FRT, attTn7::PN25tetO-gam::FRT, attλ::Ptac-uvsW^K141R^::Amp |
| CH10256 | CH4252 | MG1655 | P1 Transduction (CH4844) | yfeP::PN25-tetR::FRT, attTn7::PN25tetO-gam::FRT, ΔrnhA::FRT |
| CH10343 | CH121 | HME45 | Recombineering | ΔruvB::Kan:FRT |
| CH10346 | CH121 | HME45 | Recombineering | Δrep::Kan:FRT |
| CH10553 | CH4252 | MG1655 | P1 Transduction (CH10346) | yfeP::PN25-tetR::FRT, attTn7::PN25tetO-gam::FRT, Δrep::FRT |
| CH10681 | CH4252 | MG1655 | P1 Transduction (CH5689) | yfeP::PN25-tetR::FRT, attTn7::PN25tetO-gam::FRT, ΔruvA::FRT |
| CH10683 | CH4252 | MG1655 | P1 Transduction (CH10343) | yfeP::PN25-tetR::FRT, attTn7::PN25tetO-gam::FRT, ΔruvB::FRT |
| CH10719 | CH30 | MG1655 | P1 Transduction (CH10346) | Δrep::FRT |
| CH11320 | CH30 | MG1655 | P1 Transduction (CH4788) | ΔrecG::Kan:FRT |
| CH11322 | CH30 | MG1655 | P1 Transduction (CH5689) | ΔruvA::Kan:FRT |
| CH11324 | CH30 | MG1655 | P1 Transduction (CH10343) | ΔruvB::Kan:FRT |
| CH11326 | CH30 | MG1655 | P1 Transduction (CH4760) | ΔruvC::Kan:FRT |
| CH11328 | CH30 | MG1655 | P1 Transduction (CH5097) | ΔrecB::Kan:FRT |
| CH11330 | CH6677 | MG1655 | P1 Transduction (CH4788) | rpoB*35, ΔrecG::Kan:FRT |
| CH11332 | CH6677 | MG1655 | P1 Transduction (CH5689) | rpoB*35, ΔruvA::Kan:FRT |
| CH11334 | CH6677 | MG1655 | P1 Transduction (CH10343) | rpoB*35, ΔruvB::Kan:FRT |
| CH11336 | CH6677 | MG1655 | P1 Transduction (CH4760) | rpoB*35, ΔruvC::Kan:FRT |
| CH11338 | CH6677 | MG1655 | P1 Transduction (CH5097) | rpoB*35, ΔrecB::Kan:FRT |
| CH11578 | CH8201 | MG1655 | P1 Transduction (CH10346) | att186::dCas9::FRT, Δrep::FRT |
| CH11580 | CH8202 | MG1655 | P1 Transduction (CH10346) | att186::dCas9::FRT, Δrep::FRT |
| CH11957 | CH4252 | MG1655 | P1 Transduction (CH4788) | yfeP::PN25-tetR::FRT, attTn7::PN25tetO-gam::FRT, ΔrecG::FRT |
| CH12432 | CH30 | MG1655 | P1 Transduction (CH9246) | I-4::Kan:FRT |
| CH12434 | CH30 | MG1655 | P1 Transduction (CH4088) | ΔrecA::Kan:FRT |
| CH12430 | N/A | BW25113 | Keio Collection | ΔybfF::Kan:FRT |
| CH12438 | CH30 | MG1655 | P1 Transduction (CH5334) | ΔrecD::Kan:FRT |
| CH12442 | CH6677 | MG1655 | P1 Transduction (CH9246) | rpoB*35, I-4::Kan:FRT |
| CH12444 | CH6677 | MG1655 | P1 Transduction (CH4088) | rpoB*35, ΔrecA::Kan:FRT |
| CH12448 | CH6677 | MG1655 | P1 Transduction (CH5334) | rpoB*35, ΔrecD::Kan:FRT |
| CH12470 | CH9617 | MG1655 | P1 Transduction (CH12430) | yfeP::PN25-tetR::FRT, attTn7::PN25tetO-Gam::FRT, invD, ΔybfF::Kan:FRT |
| CH12493 | CH9617 | MG1655 | P1 Transduction (CH9964) | yfeP::PN25-tetR::FRT, attTn7::PN25tetO-MuGam::FRT, ΔseqA::FRT, invD, rpoB*35::Kan:FRT |
| CH12496 | CH9617 | MG1655 | P1 Transduction (CH6083) | yfeP::PN25-tetR::FRT, attTn7::PN25tetO-MuGam::FRT, ΔseqA::FRT, invD, ΔdiaA::Kan:FRT |
| CH13054 | CH4252 | MG1655 | Transformation | yfeP::PN25-tetR::FRT, attTn7::PN25tetO-Gam::FRT, pDS596 |
| CH13056 | CH4252 | MG1655 | Transformation | yfeP::PN25-tetR::FRT, attTn7::PN25tetO-Gam::FRT, pLS120 |
| CH13630 | CH4252 | MG1655 | Transformation | pSK762 |
| CH13632 | CH4252 | MG1655 | Transformation | pSK760 |
| CH13634 | N/A | BW25113 | Keio Collection | ΔtruB::Kan:FRT |
| CH13635 | N/A | BW25113 | Keio Collection | ΔrsmD::Kan:FRT |
| CH13692 | CH4252 | MG1655 | P1 Transduction (CH13634) | yfeP::PN25-tetR::FRT, attTn7::PN25tetO-gam::FRT, ΔtruB::FRT |
| CH13694 | CH4252 | MG1655 | P1 Transduction (CH13635) | yfeP::PN25-tetR::FRT, attTn7::PN25tetO-gam::FRT, ΔrsmD::FRT |
| CH14779 | N/A | BW25113 | Keio Collection | ΔtrmH::Kan:FRT |
| CH14780 | N/A | BW25113 | Keio Collection | ΔproQ::Kan:FRT |
| CH14783 | N/A | BW25113 | Keio Collection | Δtgt::Kan:FRT |
| CH14791 | CH5344 | MG1655 | P1 Transduction (CH4088) | ΔrecJ::FRT, ΔrecA::Kan:FRT |
| CH14792 | CH5344 | MG1655 | P1 Transduction (CH5097) | ΔrecJ::FRT, ΔrecB::Kan:FRT |
| CH14794 | CH5344 | MG1655 | P1 Transduction (CH5334) | ΔrecJ::FRT, ΔrecD::Kan:FRT |
| CH14807 | CH6677 | MG1655 | P1 Transduction (CH5333) | rpoB*35, recJ::Kan:FRT |
| CH14809 | CH4252 | MG1655 | P1 Transduction (CH14779) | yfeP::PN25-tetR::FRT, attTn7::PN25tetO-gam::FRT, ΔtrmH::FRT |
| CH14810 | CH4252 | MG1655 | P1 Transduction (CH14780) | yfeP::PN25-tetR::FRT, attTn7::PN25tetO-gam::FRT, ΔproQ::FRT |
| CH14813 | CH4252 | MG1655 | P1 Transduction (CH14783) | yfeP::PN25-tetR::FRT, attTn7::PN25tetO-gam::FRT, Δtgt::FRT |
| CH14851 | CH14807 | MG1655 | P1 Transduction (CH4088) | rpoB*35, ΔrecJ::FRT, ΔrecA::Kan:FRT |
| CH14852 | CH14807 | MG1655 | P1 Transduction (CH5097) | rpoB*35, ΔrecJ::FRT, ΔrecB::Kan:FRT |
| CH14854 | CH14807 | MG1655 | P1 Transduction (CH5334) | rpoB*35, ΔrecJ::FRT, ΔrecD::Kan:FRT |
| CH15148 | CH6224 | MG1655 | Transformation | recB^D1080A^ argA::TN10, pUA66-PsulA-GFPmut2 |
| CH15663 | N/A | FC40 | Susan Rosenberg Lab | attλ::ampRC |
| CH15691 | CH7245 | MG1655 | P1 Transduction (CH9964) | attλ::cI(ind-)-tetA, rpoB*35 I-84::FRT |
| CH15695 | CH15663 | FC40 | P1 Transduction (CH9964) | attλ::ampRC, rpoB*35 I-84::FRT |
| CH15726 | CH6677 | MG1655 | P1 Transduction (CH10346) | Δrep::FRT, rpoB*35 |

**Supplementary Table 7 – Sequencing files deposited for this study.**

| File | SRA Accession |
| --- | --- |
| Figure 1 | |
| 1_CRISPRI_WT_T0_REP1.fastq.gz | SRR32224746 |
| 2_CRISPRI_WT_T0_REP2.fastq.gz | SRR32224745 |
| 3_CRISPRI_WT_T0_REP3.fastq.gz | SRR32224734 |
| 4_CRISPRI_WT_TF_LB_REP1.fastq.gz | SRR32224723 |
| 5_CRISPRI_WT_TF_LB_REP2.fastq.gz | SRR32224712 |
| 6_CRISPRI_WT_TF_LB_REP3.fastq.gz | SRR32224701 |
| 7_CRISPRI_RECA_T0_REP1.fastq.gz | SRR32224690 |
| 8_CRISPRI_RECA_T0_REP2.fastq.gz | SRR32224679 |
| 9_CRISPRI_RECA_T0_REP3.fastq.gz | SRR32224678 |
| 10_CRISPRI_RECA_T0_REP4.fastq.gz | SRR32224677 |
| 11_CRISPRI_RECA_TF_LB_REP1.fastq.gz | SRR32224744 |
| 12_CRISPRI_RECA_TF_LB_REP2.fastq.gz | SRR32224743 |
| 13_CRISPRI_RECA_TF_LB_REP3.fastq.gz | SRR32224742 |
| 14_CRISPRI_RECA_TF_LB_REP4.fastq.gz | SRR32224741 |
| 15_CRISPRI_GAM_T0_REP1.fastq.gz | SRR32224740 |
| 16_CRISPRI_GAM_T0_REP2.fastq.gz | SRR32224739 |
| 17_CRISPRI_GAM_T0_REP3.fastq.gz | SRR32224738 |
| 18_CRISPRI_GAM_T0_REP4.fastq.gz | SRR32224737 |
| 19_CRISPRI_GAM_TF_LB_REP1.fastq.gz | SRR32224736 |
| 20_CRISPRI_GAM_TF_LB_REP2.fastq.gz | SRR32224735 |
| 21_CRISPRI_GAM_TF_LB_REP3.fastq.gz | SRR32224733 |
| 22_CRISPRI_GAM_TF_LB_REP4.fastq.gz | SRR32224732 |
| Figure 4 | |
| 23_RIFSEQ_WT_NTC_REP1_R1.fastq.gz | SRR32224731 |
| 24_RIFSEQ_WT_NTC_REP1_R2.fastq.gz | SRR32224731 |
| 25_RIFSEQ_WT_NTC_REP2_R1.fastq.gz | SRR32224730 |
| 26_RIFSEQ_WT_NTC_REP2_R2.fastq.gz | SRR32224730 |
| 27_RIFSEQ_WT_NTC_REP3_R1.fastq.gz | SRR32224729 |
| 28_RIFSEQ_WT_NTC_REP3_R2.fastq.gz | SRR32224729 |
| 29_RIFSEQ_WT_GAM_REP1_R1.fastq.gz | SRR32224728 |
| 30_RIFSEQ_WT_GAM_REP1_R2.fastq.gz | SRR32224728 |
| 31_RIFSEQ_WT_GAM_REP2_R1.fastq.gz | SRR32224727 |
| 32_RIFSEQ_WT_GAM_REP2_R2.fastq.gz | SRR32224727 |
| 33_RIFSEQ_WT_GAM_REP3_R1.fastq.gz | SRR32224726 |
| 34_RIFSEQ_WT_GAM_REP3_R2.fastq.gz | SRR32224726 |
| 35_RIFSEQ_SEQA_NTC_REP1_R1.fastq.gz | SRR32224725 |
| 36_RIFSEQ_SEQA_NTC_REP1_R2.fastq.gz | SRR32224725 |
| 37_RIFSEQ_SEQA_NTC_REP2_R1.fastq.gz | SRR32224724 |
| 38_RIFSEQ_SEQA_NTC_REP2_R2.fastq.gz | SRR32224724 |
| 39_RIFSEQ_SEQA_NTC_REP3_R1.fastq.gz | SRR32224722 |
| 40_RIFSEQ_SEQA_NTC_REP3_R2.fastq.gz | SRR32224722 |
| 41_RIFSEQ_SEQA_GAM_REP1_R1.fastq.gz | SRR32224721 |
| 42_RIFSEQ_SEQA_GAM_REP1_R2.fastq.gz | SRR32224721 |
| 43_RIFSEQ_SEQA_GAM_REP2_R1.fastq.gz | SRR32224720 |
| 44_RIFSEQ_SEQA_GAM_REP2_R2.fastq.gz | SRR32224720 |
| 45_RIFSEQ_SEQA_GAM_REP3_R1.fastq.gz | SRR32224719 |
| 46_RIFSEQ_SEQA_GAM_REP3_R2.fastq.gz | SRR32224719 |
| 47_RIFSEQ_SEQAB35_NTC_REP1_R1.fastq.gz | SRR32224718 |
| 48_RIFSEQ_SEQAB35_NTC_REP1_R2.fastq.gz | SRR32224718 |
| 49_RIFSEQ_SEQAB35_NTC_REP2_R1.fastq.gz | SRR32224717 |
| 50_RIFSEQ_SEQAB35_NTC_REP2_R2.fastq.gz | SRR32224717 |
| 51_RIFSEQ_SEQAB35_NTC_REP3_R1.fastq.gz | SRR32224716 |
| 52_RIFSEQ_SEQAB35_NTC_REP3_R2.fastq.gz | SRR32224716 |
| 53_RIFSEQ_SEQAB35_GAM_REP1_R1.fastq.gz | SRR32224715 |
| 54_RIFSEQ_SEQAB35_GAM_REP1_R2.fastq.gz | SRR32224715 |
| 55_RIFSEQ_SEQAB35_GAM_REP2_R1.fastq.gz | SRR32224714 |
| 56_RIFSEQ_SEQAB35_GAM_REP2_R2.fastq.gz | SRR32224714 |
| 57_RIFSEQ_SEQAB35_GAM_REP3_R1.fastq.gz | SRR32224713 |
| 58_RIFSEQ_SEQAB35_GAM_REP3_R2.fastq.gz | SRR32224713 |
| 59_RIFSEQ_SEQADIAA_NTC_REP1_R1.fastq.gz | SRR32224711 |
| 60_RIFSEQ_SEQADIAA_NTC_REP1_R2.fastq.gz | SRR32224711 |
| 61_RIFSEQ_SEQADIAA_NTC_REP2_R1.fastq.gz | SRR32224710 |
| 62_RIFSEQ_SEQADIAA_NTC_REP2_R2.fastq.gz | SRR32224710 |
| 63_RIFSEQ_SEQADIAA_NTC_REP3_R1.fastq.gz | SRR32224709 |
| 64_RIFSEQ_SEQADIAA_NTC_REP3_R2.fastq.gz | SRR32224709 |
| 65_RIFSEQ_SEQADIAA_GAM_REP1_R1.fastq.gz | SRR32224708 |
| 66_RIFSEQ_SEQADIAA_GAM_REP1_R2.fastq.gz | SRR32224708 |
| 67_RIFSEQ_SEQADIAA_GAM_REP2_R1.fastq.gz | SRR32224707 |
| 68_RIFSEQ_SEQADIAA_GAM_REP2_R2.fastq.gz | SRR32224707 |
| 69_RIFSEQ_SEQADIAA_GAM_REP3_R1.fastq.gz | SRR32224706 |
| 70_RIFSEQ_SEQADIAA_GAM_REP3_R2.fastq.gz | SRR32224706 |
| Supplementary Figure 6 | |
| 71_CRISPRI_WT_T0_REP1.fastq.gz | SRR32224705 |
| 72_CRISPRI_WT_T0_REP2.fastq.gz | SRR32224704 |
| 73_CRISPRI_WT_T0_REP3.fastq.gz | SRR32224703 |
| 74_CRISPRI_WT_T0_REP4.fastq.gz | SRR32224702 |
| 75_CRISPRI_WT_TF_LB_REP1.fastq.gz | SRR32224700 |
| 76_CRISPRI_WT_TF_LB_REP2.fastq.gz | SRR32224699 |
| 77_CRISPRI_WT_TF_LB_REP3.fastq.gz | SRR32224698 |
| 78_CRISPRI_WT_TF_LB_REP4.fastq.gz | SRR32224697 |
| 79_CRISPRI_WT_TF_LBcm_REP1.fastq.gz | SRR32224696 |
| 80_CRISPRI_WT_TF_LBcm_REP2.fastq.gz | SRR32224695 |
| 81_CRISPRI_WT_TF_LBcm_REP3.fastq.gz | SRR32224694 |
| 82_CRISPRI_WT_TF_LBcm_REP4.fastq.gz | SRR32224693 |
| 83_CRISPRI_REP_T0_REP1.fastq.gz | SRR32224692 |
| 84_CRISPRI_REP_T0_REP2.fastq.gz | SRR32224691 |
| 85_CRISPRI_REP_T0_REP3.fastq.gz | SRR32224689 |
| 86_CRISPRI_REP_T0_REP4.fastq.gz | SRR32224688 |
| 87_CRISPRI_REP_TF_LB_REP1.fastq.gz | SRR32224687 |
| 88_CRISPRI_REP_TF_LB_REP2.fastq.gz | SRR32224686 |
| 89_CRISPRI_REP_TF_LB_REP3.fastq.gz | SRR32224685 |
| 90_CRISPRI_REP_TF_LB_REP4.fastq.gz | SRR32224684 |
| 91_CRISPRI_REP_TF_LBcm_REP1.fastq.gz | SRR32224683 |
| 92_CRISPRI_REP_TF_LBcm_REP2.fastq.gz | SRR32224682 |
| 93_CRISPRI_REP_TF_LBcm_REP3.fastq.gz | SRR32224681 |
| 94_CRISPRI_REP_TF_LBcm_REP4.fastq.gz | SRR32224680 |

**Supplemental References**

1. Sivaramakrishnan, P., Sepúlveda, L.A., Halliday, J.A., Liu, J., Núñez, M.A.B., Golding, I., Rosenberg, S.M., and Herman, C. (2017). The transcription fidelity factor GreA impedes DNA break repair. Nature *550*, 214–218. https://doi.org/10.1038/nature23907.

2. Petrosino, J.F., Pendleton, A.R., Weiner, J.H., and Rosenberg, S.M. (2002). Chromosomal system for studying AmpC-mediated beta-lactam resistance mutation in Escherichia coli. Antimicrob Agents Chemother *46*, 1535–1539. https://doi.org/10.1128/AAC.46.5.1535-1539.2002.
